# Upper Bound on the Mutational Burden Imposed by a CRISPR-Cas9 Gene-Drive Element

**DOI:** 10.1101/2023.11.28.569142

**Published:** 2023-11-29

**Authors:** Michael S. Overton, Sean E. Guy, Xingsen Chen, Alena Martsul, Krypton Carolino, Omar S. Akbari, Justin R. Meyer, Sergey Kryazhimskiy

**Affiliations:** 1 Department of Ecology, Behavior and Evolution, School of Biological Sciences, University of California San Diego, La Jolla, CA 92093; 2 Current address: Bionano Genomics, San Diego, CA 92121; 3 Current address: Department of Entomology, University of Arizona, Tucson, Arizona, USA; 4 Current address: Illumina Inc., San Diego, CA 92122; 5 Department of Cell and Developmental Biology, School of Biological Sciences, University of California San Diego, La Jolla, CA 92093, USA

## Abstract

CRISPR-Cas9 gene drives (CCGDs) are powerful tools for genetic control of wild populations, useful for eradication of disease vectors, conservation of endangered species and other applications. However, Cas9 alone and in a complex with gRNA can cause double-stranded DNA breaks at off-target sites, which could increase the mutational load and lead to loss of heterozygosity (LOH). These undesired effects raise potential concerns about the long-term evolutionary safety of CCGDs, but the magnitude of these effects is unknown. To estimate how the presence of a CCGD or a Cas9 alone in the genome affects the rates of LOH events and de novo mutations, we carried out a mutation accumulation experiment in yeast *Saccharomyces cerevisiae*. Despite its substantial statistical power, our experiment revealed no detectable effect of CCGD or Cas9 alone on the genome-wide rates of mutations or LOH events, suggesting that these rates are affected by less than 30%. Nevertheless, we found that Cas9 caused a slight but significant shift towards more interstitial and fewer terminal LOH events, and the CCGD caused a significant difference in the distribution of LOH events on Chromosome V. Taken together, our results show that these genetic elements impose a weak and likely localized additional mutational burden in the yeast model. Although the mutagenic effects of CCGDs need to be further evaluated in other systems, our results suggest that the effect of CCGDs on off-target mutation rates and genetic diversity may be acceptable.

## Introduction

The CRISPR-Cas9 based gene drives (CCGDs) are synthetic genetic elements that can rapidly spread in sexual populations through super-mendelian inheritance (Bier 2021). The ability to encode various functions within CCGDs promises to give us an unprecedented degree of control over wild populations (Champer, Buchman, and Akbari 2016). For example, by encoding a *Plasmodium*-disrupting peptide within a CCGD, we may be able to eliminate the spread of malaria, or a CCGD encoding drought resistance may allow us to prevent the extinction of populations vulnerable to climate change (Wang and Jacobs-Lorena 2013; Champer, Buchman, and Akbari 2016; Li et al. 2008). However, large-scale deployment of CCGDs in the wild faces several significant biological, as well as ethical, challenges (Rode et al. 2019; Oberhofer, Ivy, and Hay 2018; Esvelt et al. 2014). Evolution presents a major biological challenge, which can manifest as two distinct problems (Rode et al. 2019). The first, short-term, problem is that certain mutations can arise in the CCGD element itself, abolishing its activity. Given that CCGD carriage often comes with a fitness cost, such loss-of-function mutations would be favored by selection and would result in gene drive-resistant populations (Unckless, Clark, and Messer 2017; Beaghton et al. 2019; Champer et al. 2017). This problem is widely recognized, and progress has been made in mitigating it (Marshall and Akbari 2018; Marshall et al. 2017; Kandul et al. 2021). The second problem is that Cas9 has off-target effects, which could increase the incidence of new mutations as well as loss of heterozygosity (LOH) across the genome, potentially altering long-term evolutionary trajectories of treated populations. However, the magnitude of these effects and therefore their evolutionary significance are unknown (Rode et al. 2019).

CCGDs vary in their design and complexity, but every CCGD contains a guide RNA (gRNA) that targets a particular genomic sequence (“the target allele”) and a Cas9 endonuclease (Gantz and Bier 2015; Champer, Buchman, and Akbari 2016). In addition, CCGDs can also contain a genetic “payload” that produces a desired phenotype. The drive construct must be flanked by regions of homology to the regions around the target allele. When an engineered organism homozygous for the CCGDs mates with a wildtype (WT) individual carrying the target allele, the fusion of gametes brings the drive and the target sequences together into the same cell. The Cas9/gRNA complex cuts the target allele, and the entire CCGD can be copied in its place by the homology directed repair (HDR), thereby converting an initially heterozygous offspring into one homozygous for the CCGD allele. Through this process, CCGDs can achieve up to 98% inheritance, rapidly fixing the desired phenotype in the target population (Gantz et al. 2015; Adolfi et al. 2020).

The presence of a CCGD in the genome can change the rates and types of genome-wide mutations via several known as well as possibly other, as of yet unknown, mechanisms (Cradick et al. 2013; Fu et al. 2013; Hsu et al. 2013; Lin et al. 2014; Pattanayak et al. 2013; Tsai et al. 2017; Newton et al. 2019). The best understood mechanism is template promiscuity whereby the Cas9/gRNA complex binds and cuts DNA sequences that are similar but not identical to the target (Fu et al. 2013; Hsu et al. 2013; Lin et al. 2014; Boyle et al. 2021). These off-target dsDNA cuts are repaired by HDR or non-homologous end-joining (NHEJ), leading to LOH and indel mutations, respectively, at loci other than the target (Cradick et al. 2013; Fu et al. 2013). Off-target mutations may also be generated because the Cas9-gRNA complex bound to DNA might interfere with normal replisome progression. Importantly, such binding transiently occurs not only at the sequences similar to the target but also at random PAM sites (Sternberg et al. 2014). Moreover, the Cas9 protein alone has a high, non-specific affinity for DNA (Sternberg et al. 2014), which can potentially introduce additional mutations at non-PAM associated sites through the same interference mechanism. These additional mutations and LOH events could have unpredictable evolutionary consequences. For example, some LOH events could resolve hybrid incompatibilities and improve fitness (Smukowski Heil et al. 2017; James et al. 2019; Mandegar and Otto 2007). Perhaps more likely, by reducing genetic diversity and exposing recessive deleterious alleles, LOH events could potentially lead to population decline, particularly in species that are already endangered (Wernberg et al. 2018; Frankham 1995). However, the severity of these long-term evolutionary consequences depends on how strongly CCGDs affect the rates of mutations and LOH events, on the type of these events, and on their distribution along the genome. Thus, to quantify evolutionary risks associated with CCGDs, we need to measure how CCGDs affect the rates and the distributions of these events.

To address this problem, we carried out a mutation accumulation (MA) experiment in the yeast *Saccharomyces cerevisiae*, an established system for testing CCGDs (DiCarlo et al. 2015). A major advantage of MA experiments over other higher-throughput assays is that they allow us to detect the effects of gene drives caused by known as well as unknown molecular mechanisms. Using yeast allows us to achieve two further goals. First, by carrying out the MA experiment in a strain that is heterozygous at about 40,000 sites throughout the genome, we are able to detect genome-wide LOH events with high resolution (Yin and Petes 2013; Zheng et al. 2016; Sui et al. 2020; Loeillet et al. 2020; Dutta, Dutreux, and Schacherer 2021; Pankajam et al. 2020; Dutta et al. 2017). Second, using yeast allows us to maintain hundreds of independent MA lines for hundreds of generations, which maximizes our statistical power to detect changes in the LOH and mutation rates due to the presence of a CCGD or a Cas9 alone in the genome.

## Results

### A priori analysis of statistical power and the design of the mutation accumulation experiment

**Figure 1.**
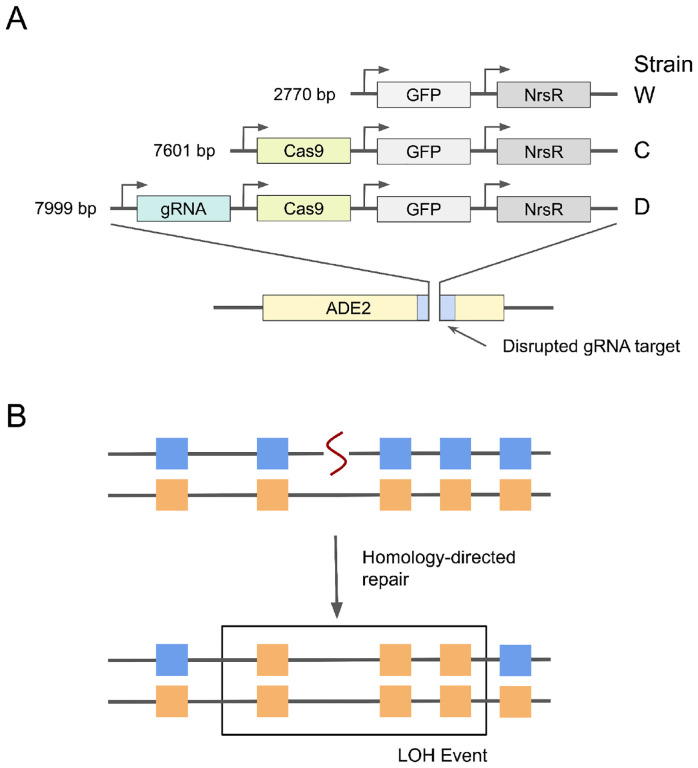
Genetic constructs used in this study and the schematic of how LOH events are detected. **A.** Design of constructs for the three founder strains. All constructs were integrated into the same location in the ADE2 locus at Chr XV. **B.** Detection of LOH events. Our founder strains are diploid hybrids heterozygous at about 40,000 marker sites across the genome (squares of different colors). If a dsDNA break (squiggly line) is repaired by homology directed repair, it causes loss of heterozygosity at nearby marker sites, which can be detected in whole-genome sequencing data.

We set out to measure how the long-term presence of a CCGD element or a Cas9 alone in the genome alters the LOH and mutation rates in yeast. To this end, we carried out mutation accumulation (MA) experiments in three diploid *S. cerevisiae* hybrid “founder” strains, which we refer to as D, C and W (Figure 1A). All three founders were derived from a cross between the haploid laboratory strain BY and the vineyard isolate RM, which differ by over 40,000 genome-wide SNPs and small indels (Bloom et al. 2013). Thus, all three founders are heterozygous at these ~40,000 positions, which corresponds to an average distance of 300 bp between markers, allowing us to detect LOH events with relatively high resolution (Figure 1B and Refs. (Dutta, Dutreux, and Schacherer 2021; Pankajam et al. 2020; Yin and Petes 2013; Zheng et al. 2016)).

The founder strains differ from each other by the type of construct that we integrated into the *ADE2* locus (Methods; Figure 1A). The W strain is the wild-type control which carries two copies of the integration cassette without any gene-drive components. The C strain carries two copies of the constitutively expressed Cas9 gene. We designed this strain so as to look for possible mutagenic effects of a Cas9 protein that is not associated with a gRNA, something that can occur for example if the Cas9 and gRNA expression levels are not perfectly matched (Hsu et al. 2013). Finally, the D strain carries two copies of a constitutively expressed CCGD with the gRNA targeting *ADE2*. Since the *ADE2* gene is disrupted by the CCGD cassette, the target sequence is absent from the D strain genome, such that any cut performed by the Cas9/gRNA complex would be off-target. This strain was designed to simulate a gene drive that had successfully fixed in a population, and is now permanently integrated into the species’ genome.

Our a priori expectation was that the main side-effect of the presence of a CCGD element in the genome would be an HDR-driven increase in the rate of LOH events at off-target sites. We thus sought to design an MA experiment that would have sufficient statistical power to detect moderate increases in this rate. To estimate statistical power of MA experiments that are practically feasible, we modeled the accumulation of LOH events in each MA line as a Poisson process. We found that, if each wildtype MA line accumulates on average 12 LOH events, the probability of detecting a 15% increase in the LOH rate with 100 MA lines per founder at *P*-value of 0.01 would be 80.4%. Given that the previously measured rate of LOH events averages 2.5×10^−2^ (range from 3.6×10^−3^ to 4.7×10^−2^) per genome per generation (Sui et al. 2020; Pankajam et al. 2020), we reasoned that we would achieve this statistical power by propagating our MA lines for 750 cell divisions.

**Figure 2.**
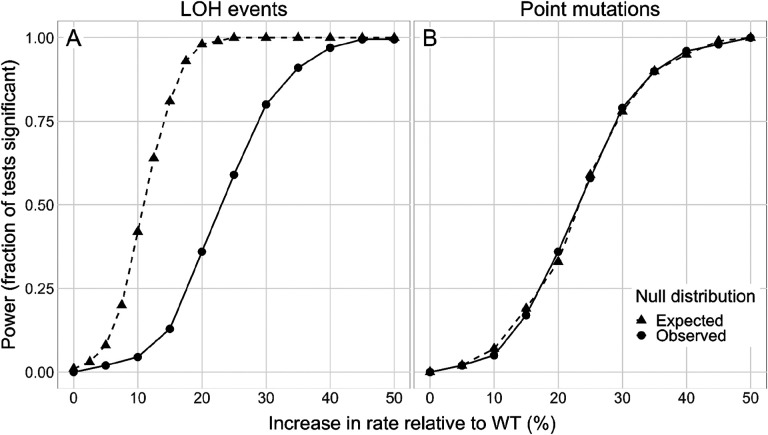
Estimates of statistical power to detect differences in LOH and mutation rates in our mutation accumulation experiment. **A.** The expected and observed power to detect a CCGD- or Cas9-associated increase in the LOH rate, at *P*-value 0.01 (see text for details). Power is defined as the fraction of simulations where a statistically significant effect was detected. **B.** The expected and observed power to detect a CCGD- or Cas9-associated increase in the point mutation rate, at *P*-value 0.01 (see text for details).

Guided by our a priori estimates, we established 95 MA lines per founder strain, for a total of 285 MA lines, and propagated them using a standard MA protocol (Methods). Briefly, for each MA line, we picked a random colony from a previous growth cycle, streaked it to single cells and let the resulting colonies grow for 48 hours. Assuming that each growth cycle corresponds to 20 cell divisions, we predicted a typical MA line would accumulate 12 LOH events after 38 cycles. To account for variation among LOH-rate estimates across studies, we propagated our MA lines for 43 cycles. After the experiment was completed, we counted the number of cells per colony after 48 hours of growth and estimated that each MA line in fact underwent approximately 800 cell divisions (Methods). Black lines in Figure 2 show the a priori expected power curves to detect LOH and mutation rate increases in our MA experiment. They suggest that our MA experiment should have sufficient statistical power to detect moderate increases in the LOH rate triggered by the presence of a CCGD or the Cas9 gene in the genome.

After the experiment was completed, we sequenced the full genomes of all founder and end-point clones to a median depth of 22x. We excluded 17 clones from further analysis due to low coverage or traces of cross-contamination during library preparation (Methods). In addition, we found that the ancestors of two D MA lines were apparently triploid, compromising our ability to detect new LOH events (Methods). Excluding these MA lines from LOH analysis, we retained 64 D, 84 C, and 75 W end-point clones. We then used bowtie2 and GATK software, along with a series of custom filters, to call the genotype (BY/BY, RM/RM or BY/RM) of each end-point clone at each marker site and to identify new mutations in each end-point clone (see Methods for details).

Random mutations can deactivate the Cas9 and/or the gRNA genes. Given the mutation rates in yeast and the sizes of our constructs, we expected to find between 0.7 and 2.3 new mutations within the constructs in our entire MA experiment (see Methods). To test this expectation, we aligned reads and called new variants for each end-point W, C, and D clone with the associated CCGD element reference sequence (Methods). We found a total of three SNPs and two indels within the constructs among all end-point clones, all of which were heterozygous and in independent clones. Only two of these mutations hit drive components, a non-synonymous SNP (A299T) in the Cas9 gene of a C clone and an indel at the 3’ end of the gRNA promoter in a D clone (Supp. Fig. 1A). Although the number of mutations is somewhat higher than expected, they are rare enough to have negligible influence on our LOH and mutation rate estimates even if all the detected mutations fully deactivated the Cas9 and/or gRNA.

Based on this analysis, we expected the vast majority of end-point mutants to have fully active constructs. To confirm this expectation, we picked six end-point D clones and experimentally measured the activity of their CCGD elements. To do so, we sporulated these clones, crossed their haploid offspring with a haploid strain containing the *ADE2* allele targeted by the drive gRNA and observed a change in colony color, as expected for an active CCGD (Supp. Fig. 1B; see Methods for details).

In summary, we designed and carried out an MA experiment with a reasonable statistical power to detect possible mutagenic effects of gene drives and/or Cas9 alone. Furthermore, all available evidence strongly suggests that the Cas9 and/or the gRNA genes remained active as expected during this experiment in the vast majority of our D and C MA lines.

### An improved method for detecting LOH events and the overall rate of LOH events

Detection of new LOH events critically hinges on the accuracy of genotype calls. We found that the standard practice of using either only the BY or only the RM reference genome for read mapping and genotype calling (Sui et al. 2020; Loeillet et al. 2020; Pankajam et al. 2020; Dutta, Dutreux, and Schacherer 2021) produces substantial allele-specific biases (Supp. Fig. 2A). Moreover, this mapping bias leads to a bias in the estimate of LOH events toward the reference sequence homolog (Supp. Fig. 2B). To remedy this problem, we designed a procedure for merging and reconciling the genotype call sets obtained against the two reference genomes. The resulting reconciled procedure is symmetric with respect to the two reference genomes and produces no detectable allele-specific biases (Supp. Fig. 2). This procedure retained on average 32,188 (range: 23,910 – 39,189) markers with high-confidence genotype calls per clone, corresponding to one marker per 375 bp on average. We found that an average of 1286 (range: 8 – 7216) markers became homozygous in a typical end-point clone, indicating that LOH events happen and we are able to detect them.

Even though our genotype calling procedure is unbiased, it is not error-free. In particular, markers that are in fact heterozygous in an end-point clone may be falsely called homozygous, and vice versa. The former errors are expected to be more common because tens of thousands of markers per end-point clone are in fact heterozygous and only a fraction of them are homozygous as a result of LOH events. Therefore, false homozygous calls in end-point clones can substantially inflate our LOH rate estimates. Previous studies mitigated this problem by discarding apparent LOH events that are supported by less than some threshold number (2 to 10) of adjacent markers (Sui et al. 2020; Loeillet et al. 2020; Pankajam et al. 2020; Dutta, Dutreux, and Schacherer 2021). However, these thresholds have not been statistically justified. As a result, requiring two or more adjacent markers may undercount LOH events with short tracks of homozygosity and/or those that occur in marker-poor genomic regions. We therefore opted to avoid this filter. Instead, we developed an LOH-rate estimate that includes all homozygous markers but corrects for the false homozygous calls.

We first defined a putative LOH event as a region spanning one or more adjacent markers heterozygous in the founder and homozygous in the end-point clone (Figure 1B; Methods). DNA strands can spontaneously rearrange during HDR, resulting in complex conversion events with intervening regions of homozygosity and heterozygosity, each spanning up to 10 kb (Lee et al. 2009; Yin and Petes 2013; Zheng et al. 2016; Sui et al. 2020). To account for this effect, we first estimated the boundaries of each putative LOH event as the positions half-way between the initial/final homozygous marker and the preceding/following heterozygous marker. We then merged putative LOH tracts in the same clone and chromosome whose boundaries were separated by less than 10 kb into single LOH events. If these complex events contained LOH tracts from both homologs, the donor homolog of the longest tract was attributed to the entire event. We found a total of 1643 putative LOH events across all our MA lines (a median of 7 per end-point clone; Supp. Fig. 3), supported by a total of 263,979 markers across all clones. 491 (29.9%) of these putative LOH events were supported by a single homozygous marker.

We next estimated the rate of false homozygous calls, based on the frequency of markers (falsely) called homozygous in our founders (Methods). We found this rate to be 8.3×10^–5^ per marker, which suggests that, on average, 3 homozygous markers discovered in a typical end-point clone are actually false positives. Given this estimate, we expect 560 out of 263,979 of apparently homozygous markers to be in fact heterozygous. The LOH events supported by multiple markers are more likely to represent true LOH events than those supported by only one or two markers. Therefore, we marked all 491 LOH events supported by a single marker as errors. We then randomly distributed the remaining 69 expected false homozygous calls among the 153 LOH events supported by two markers. This resulted in on average eight (5.2%) of these events being false positives. We therefore conclude that LOH events supported by two or more markers are overwhelmingly likely to represent true LOH events. Thus, our procedure produced a threshold that turns out to be the same as in some previous studies (Pankajam et al. 2020; Dutta, Dutreux, and Schacherer 2021). However, we note that our procedure could produce a different threshold depending on the parameters of the experiment, such as the number of sequenced clones and coverage.

After this filtering, we retained 1152 LOH events that are likely to be true (a median of 5 per line), which we use for all of the subsequent analyses, unless otherwise noted. This yields an overall estimate of the LOH event rate of 6.4×10^–3^ per genome per generation across all clones. On average, 2.6% of the genome was affected by at least one LOH event in our MA experiment. These results are in good agreement with previous work (Pankajam et al. 2020; Dutta, Dutreux, and Schacherer 2021; Sui et al. 2020; Dutta et al. 2017).

### No evidence for allele-specific biases among LOH events

Previous studies have reported that LOH events in yeast can exhibit allele-specific biases, in the sense that heterozygosity is lost in favor of one of the alleles more often than the other (Dutta, Dutreux, and Schacherer 2021; Dutta et al. 2017; Loeillet et al. 2020). Specifically, Dutta et al., 2017 and Loeillet et al., 2020 observed that 52–58% of LOH events in some BY hybrids are converted to the BY homolog, while Dutta et al., 2021 found some hybrid strains with LOH events converting to one parent 80% of the time. These studies attributed the biases to recessive lethal alleles, allele-specific chromosome instability, and/or epigenetic effects. However, since these studies relied on mapping and genotype calling procedures that were based on the BY reference genome alone, it is also possible that some of these biases are explained by mapping and/or genotype-calling artifacts. Therefore, we set out to verify that allele-specific biases in LOH events persist when genotypes are called using our fully symmetric procedure.

We found that 594 out of 1152 (51.5%) of the high-confidence LOH events resolved towards the BY allele, well within the binomial expectation (*P* = 0.30, Supp. Fig. 2B). Similarly, we found no evidence of allele-specific biases when we consider three founders separately (Table 1; *P* = 0.643 for W, *P* = 0.083 for C, and *P* = 0.781 for D; Table 1, Supp. Fig. 5). These results suggest that the LOH events do not exhibit allele-specific biases, either in the presence or in the absence of CCGD or Cas9.

### Upper bound on the increase in the genome-wide LOH-rate imposed by CCGD or Cas9

**Figure 3.**
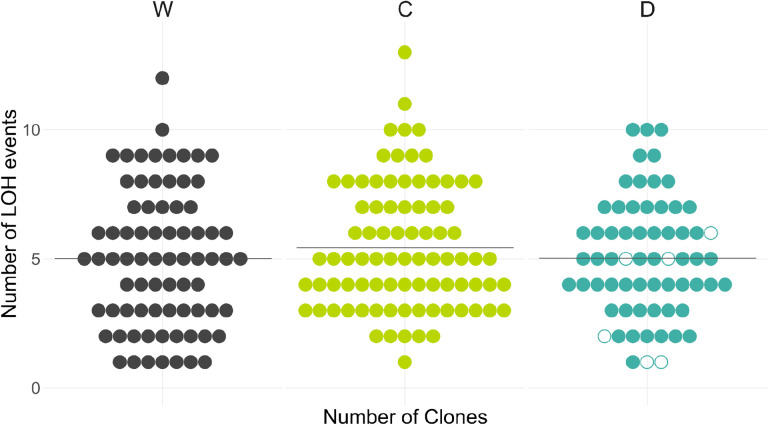
Distributions of the LOH events among end-point clones. The number of the LOH events per clone, separated by founder. Hollow circles indicate clones tested for CCGD activity (see Supp. Fig. 1). Horizontal lines indicate the mean number of LOH events per clone.

We next examined whether the accumulation LOH events differed between clone groups descended from the three strains. We identified 376, 456, and 322 LOH events in W, C, and D clones, respectively. Neither C nor D clones differed significantly from the W clones in terms of the mean number of LOH events per clone (*P* = 0.337 and *P* = 0.853, respectively; permutation test; Fig. 3, Table S1). The mean fractions of the genome affected by LOH events (2.9%, 2.3% and 2.6% in the W, C, D clones D, respectively) were also statistically indistinguishable (*P* = 0.08 and *P* = 0.46 for C vs W and D vs W comparisons respectively, permutation test).

Given this absence of a detectable effect of the CCGD or Cas9 on the overall LOH rate, we set out to estimate the upper bounds on these effects, given the power of our experiment. We expected that our power to detect differences in LOH rates was lower than anticipated a priori because we ended up using fewer MA lines in our analysis and because LOH events may not be Poisson-distributed (Supp. Fig. 4). Using a permutation-based approach (Methods), we found that the chance of detecting a 15% LOH-rate increase at the *P*-value 0.01 in our data was in fact about 13% instead of the anticipated 80.4%. Nevertheless, our experiment gives us power close to 100% to detect the effect sizes above 45% and substantial power to detect the effect size above 30% (Figure 2A). Therefore, our results suggest that the presence of a CCGD or the Cas9 protein alone increases the LOH rates in yeast likely by less than 30% and almost certainly by less than 45%.

### Cas9 increases the proportion of interstitial LOH events

Double-stranded DNA breaks can be repaired by two distinct HDR mechanisms, resulting in the so-called “interstitial” and “terminal” LOH events (iLOH and tLOH). If the break is due to direct disruption of DNA bonds, such as with UV radiation or nucleases, repair usually follows the gene conversion pathway, in which a donor homolog is replicated along the internally resected portion of the broken chromosome, resulting in an iLOH (Hum and Jinks-Robertson 2017; Mehta and Haber 2014). If the repair initiation is delayed, or if one of the chromosome fragments is not intact, the entire terminal portion of the broken chromosome is replaced by a copy of the donor homolog, from upstream of the break area to the missing chromosome end, resulting in a tLOH (Malkova et al. 2001; Malkova, Ivanov, and Haber 1996). tLOH events are associated with replication stalling during S-phase and may occur due to replisome collisions with protein-DNA complexes (Shyian and Shore 2021; Labib and Hodgson 2007). Even without affecting global breakage rates, the DNA-binding activity of Cas9, alone or in complex with a gRNA, could interfere with the DNA processing during HDR or produce replication fork stalling, and thus change the relative frequency of the two LOH types (Sternberg et al. 2014). We next tested whether this was the case for genome-wide LOH events in our MA experiment.

We found that 764 out of 1152 (66.3%) of all LOH were iLOH with the remaining 388 (33.6%) being tLOH, consistent with previous estimates (Dutta, Dutreux, and Schacherer 2021; Sui et al. 2020). The relative proportions of tLOH and iLOH events were statistically indistinguishable between D and W strains (P = 0.9374; Fisher’s exact test). However, among the C strains, 328 out of 456 (71.9%) of LOH events were iLOH, significantly more than among the W strains (*P* = 0.0046; Fisher’s exact test; Table 1; Supp Fig. 6). Thus, the presence of the Cas9 protein in the cell appears to alter the probabilities with which different dsDNA break repair mechanisms are initiated without substantially increasing the dsDNA break rates themselves.

Interactions between Cas9 to DNA could also disrupt key repair processes such as strand resection, strand invasion, or polymerase progression, leading to changes in the length distributions of ensuing LOH events. In order to test this, we compiled the lengths of all LOH events in our dataset, which varied by nearly five orders of magnitude (range = 23bp-1.5Mb; median = 10,981bp). Since the C strain was significantly enriched for iLOH events, we first separated events by type, and found distinct length distributions between iLOH and tLOH events, as observed previously (Dutta, Dutreux, and Schacherer 2021). Interstitial events were significantly shorter (median = 4,668bp) than terminal events (median = 180kb; P = 0.0001, Wilcoxon rank-sum test), and appeared to follow a bimodal distribution (Supp. Fig. 11). When comparing the length distributions of each LOH type between the W strain and the C and D strains, we did not observe any significant differences (*P* > 0.10, Wilcoxon rank-sum test). This suggests that Cas9 does not interfere extensively with the progression of dsDNA break repair.

### Evidence that CCGD and Cas9 alone alter local LOH event rates

We have shown so far that the presence of the CCGD or the Cas9 gene in the genome does not detectably alter the global rate of LOH event accumulation or their allele-specific biases, but may affect the rate of iLOH events. Given that the CCGD off-target activity is sequence specific, it is possible that CCGD or the Cas9 protein strongly affect LOH rates only in certain regions of the genome and that such local effects are missed when the data are pooled across the whole genome. Thus, we next asked whether founders differ from each other in terms of LOH rates chromosome by chromosome or in terms of the distributions of LOH events within chromosomes.

**Figure 4.**
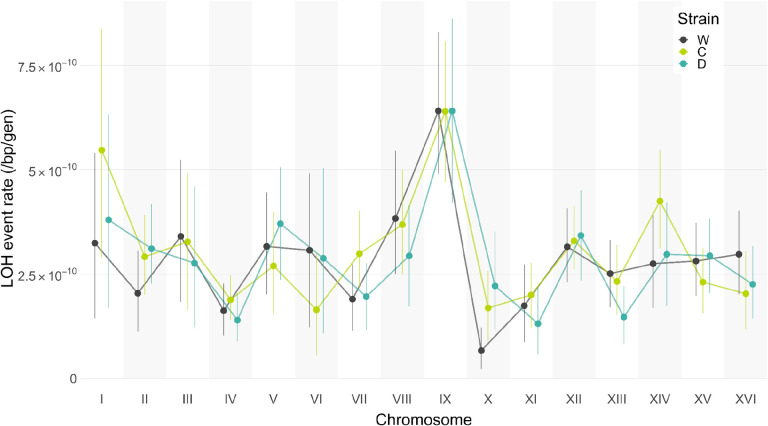
Distributions of LOH rates across chromosomes. LOH event rates for the three founders are plotted for each of the 16 yeast nuclear chromosomes. Filled circles show the mean and vertical bars indicate the 95% bootstrap confidence intervals.

We stratified the LOH events by chromosome and estimated the rate of LOH events per unit chromosome length (Supp. Table 1). We found that the per base-pair LOH rates vary by 4.3-fold across chromosomes; the highest rate was 6.41×10^–10^ per bp per generation on Chr IX, and the lowest 1.50×10^–11^ per bp per generation on Chr X (Figure 4, Supp Table 1). Overall, the distribution of LOH events across chromosomes in the C and D clones were statistically indistinguishable from that in the W clones (*P* = 0.357, *P* = 0.291, respectively, χ^2^ test). Similarly, we find no statistical differences when we look at iLOH and tLOH events separately (Supp. Fig. 7).

We next looked for possible differences in the rates of LOH events among strains at sub-chromosomal resolution. There are two potentially interesting metrics to consider. Given the expected nuclease activity of Cas9, we have so far analyzed the LOH event rate as a proxy for the rate of dsDNA breaks. We can apply this reasoning to finer scales by estimating how often LOH-inducing dsDNA breakpoints occur at a given base pair in the genome. We refer to this metric as the breakpoint rate. The presence of a CCGD or Cas9 gene could also affect the rate at which particular heterozygous sites undergo LOH, for example by interfering with break repair at particular genomic regions. Therefore, we also estimated the LOH conversion rate per bp, that is the rate at which a given base pair in the genome would convert from the heterozygous to a homozygous state (Dutta, Dutreux, and Schacherer 2021; Sui et al. 2020).

To pinpoint the locations of the LOH-inducing dsDNA breakpoints, we estimated the boundaries of each LOH event as the midpoint between the two adjacent markers surrounding each boundary (Methods). Then, for each iLOH, we estimated the position of the causal dsDNA break as the midpoint between its two boundaries (Hum and Jinks-Robertson 2017); for each tLOH, we used its interior boundary as an estimated position of the causal dsDNA break. In order to analyze the positional distribution of breakpoints, we applied a sliding window function, which calculated the breakpoint rate for 50kb windows with a 5kb shift along each chromosome.

**Figure 5.**
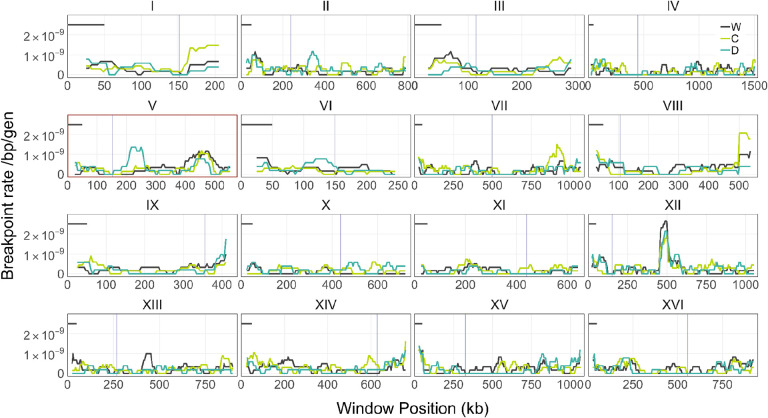
Distribution of LOH-inducing breakpoint rates across the genome. Each line shows the breakpoint rate estimated per 50 kb sliding window with a 5 kb step size (see Methods for details). The y-axis scale has been truncated to show subtle changes in breakpoint rate, and so cuts off the breakpoint hotspot at Chr IX (see Supp. Fig. 8 for the full scale figure). Line segments are scale bars of the 50kb window. Red outline indicates the chromosome with a significantly different breakpoint distribution between the D and W strains.

The dsDNA breakpoint rate in 50kb sliding windows for each founder is shown in Figure 5. Breakpoints are evenly distributed across positions for most chromosomes, aside from the previously mentioned rRNA gene array on Chr XII and a notable breakpoint hotspot at Chr IX (Supp. Fig. 8). In all three strains, LOH events were significantly enriched at a relatively small region on the right arm of Chr IX, suggesting a DNA breakage hotspot (Hegazy uniformity test, P < 0.001, Methods). If we separate iLOH and tLOH events, we see that this LOH hotspot appears to be driven primarily by gene conversions rather than BIR repair (Supp Fig 9). Since the affected region is within 10kb of the chromosome end, we may suspect that these are actually tLOH events. However, those that are marked as iLOH tracts are followed by multiple distal heterozygous markers, supporting their categorization. Numerous iLOH events concentrated in this region would suggest the presence of recombinogenic elements such as Ty elements, tRNA genes, or inverted repeats, all of which can increase the occurrence of gene conversions (Song et al. 2014; Rosen et al. 2013). However, we found only a single Ty element within 10kb of this hotspot. Interestingly, this particular region of increased activity has not been observed in previous studies and may reflect strain-specific effects in our experiment.

We next asked whether the distributions of LOH-inducing dsDNA breakpoints on each chromosome differ between D and C clones compared to the W clones (Figure 5). We observed some apparent differences when we plotted how the breakpoint rate changes across the genome in a 50 kb sliding window (e.g. on Chr I, II, V, VIII, XIV). However, since we have only on average 1.6 LOH-inducing breakpoints per window per strain, we do not have enough statistical power to distinguish between strains at this resolution. We therefore compared the cumulative distributions of breakpoint positions in the D and C strains against that of the W strain. This type of comparison increases our power at the expense of our ability to precisely identify the genomic locations where the strains differ. We found that the distribution of breakpoints on Chr V in the D clones differs significantly from that in the W clones (Benjamini-Hochberg adjusted *P* = 0.026; Wasserstein test, see Methods).

While we do not have sufficient statistical power to ascertain the precise genomic locations where the D-clones differ from W-clones, we found that the D-clones have an unusually high concentration of LOH-inducing breakpoints between positions 180 kb and 271 kb on Chr V. Specifically, we identified 9 breakpoints among the 64 D-clones (0.141 per clone) but only one breakpoint among 75 W-clones (0.013 per clone) and only three breakpoints among 84 C-clones (0.036 per clone). We analyzed the DNA-sequence statistics within this region looking for evidence that this apparent difference is driven by off-target CCGD activity, but found none (Methods). We further discuss these observations in the Discussion.

Local increases in the breakpoint rate did not always translate into increases in the rate of LOH conversion, likely due to heterogeneity in the resulting LOH lengths (Supp. Fig. 10). For example, the numerous long tLOH events on the right arm of Chr XII produced a high conversion rate in that region, while the many short LOH events at the tail of Chr IX did not. Conversion rates tended to diverge among the strains primarily at the ends of chromosome arms, with lower rates and less divergence near centromeres. Therefore, to test whether there were any significant local increases in conversion rates, we divided each chromosome into thirds, and calculated the mean conversion rate of each founder. We did not detect any significant differences in conversion rates between the D and W strains or the C and W strains across all chromosome regions, even for the region of significantly concentrated D strain breakpoints at Chr V (Benjamini-Hochberg adjusted *P* > 0.59, permutation test).

Taken together, these results suggest that the presence of a CCGD or Cas9 alone in the genome may increase local DNA break rates in some regions of the genome, but these regions may be difficult to predict a priori.

### Upper bound on the increases in the genome-wide mutation rate imposed by CCGD or Cas9

In addition to affecting LOH rates, the presence of the CCGD or Cas9 protein could also increase the rate of new mutations. To test this, we identified new mutations in our end-point clones (Methods). We found a total of 1322 single-nucleotide mutations (SNMs) and 253 small indels (< 50 bp) across all end-point clones, yielding mutation rate estimates of 3.2×10^–10^ SNMs and 6.1×10^–11^ small indels per base-pair per generation. While the SNM rate was consistent with previous work (Lang and Murray 2008; Zhu et al. 2014; Sui et al. 2020; Pankajam et al. 2020), the indel rate was elevated by 8–16 fold compared to previous estimates (Liu and Zhang 2019; Zhu et al. 2014; Sui et al. 2020). This discrepancy may be due strain-specific mutation rates, as these previous estimates were performed in homozygous diploid strains, whereas our estimate more closely resembles that of another study (6.5×10^–11^) in a BY×SK1 hybrid strain (Loeillet et al. 2020).

**Figure 6.**
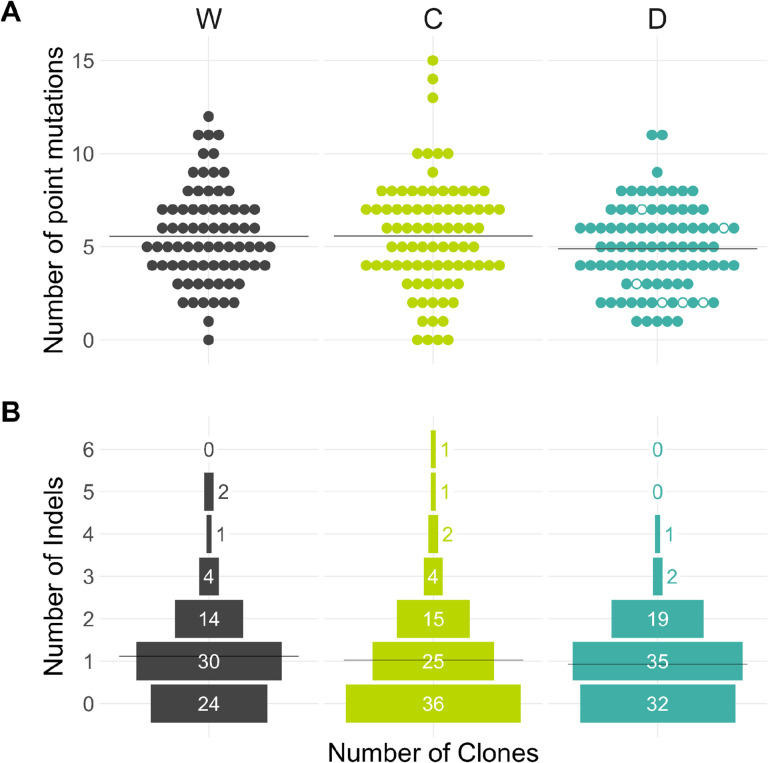
Distributions of SNMs and indels among end-point clones. **A.** Number of point mutations per clone, separated by strain. Hollow circles indicate clones tested for drive activity. **B.** Distribution of the number of indels in end-point clones. Horizontal lines indicate the mean number of mutations per clone for each strain and mutation type.

A total of 435, 469, and 418 SNMs and 83, 86, and 84 indels were found in the D-, C-, and W-strains, respectively (Table 1, Figure 6). The distribution of SNMs and indels did not differ significantly between D- and W-strains (*P* = 0.072 for SNMs and P = 0.644 for indels, permutation test) or between C- and W-strains (*P* = 0.99 for SNMs and P = 0.245 for indels). Thus, analogous to the LOH rate analysis, we estimated the statistical power of our experiment to detect differences in mutation rates among strains (Methods). We found that the likelihood of detecting a 30% increase in mutation rate is 80%, and a 50% increase is near 100% (Figure 2D). Therefore, we conclude that presence of an active CCGD or the Cas9 protein alone increases point mutation rates in yeast likely by less than 30% and almost certainly by less than 50%. We also found no evidence that local mutation rates differ between founders at the chromosome and sub-chromosome resolution (see Methods for details). Thus, the additional burden of new point mutations and indels imposed by the presence of a CCGD or a Cas9 gene is likely negligible compared to the magnitude of natural mutation-rate variation along the genome (about six-fold, (Lang and Murray 2011)) or between yeast strains (about five-fold, (Gou, Bloom, and Kruglyak 2019; Pankajam et al. 2020)).

## Discussion

In this study, we investigated how the presence of a CRISPR-Cas9 gene drive (CCGD) element or the Cas9 gene in the genome affects the rates of LOH events, new point mutations and indels in yeast. To carry out these analyses, we developed a new method for detecting LOH events which is free of mapping biases characteristic of previous methods. Importantly, LOH events inferred by our method do not exhibit allele-specific biases that were reported previously.

Interestingly, we found no evidence that the overall LOH or mutation rates were elevated in strains carrying the CCGD or Cas9 gene. Given the large size of our study, the absence of statistically significant effects strongly suggests that these effects are relatively small. Specifically, we estimated that the presence of a CCGD or the Cas9 protein alone increases the LOH and mutation rates likely by no more than 30%. By comparison, previous studies found up to a 50-fold difference in LOH event rates among yeast hybrid strains (Dutta, Dutreux, and Schacherer 2021; Sui et al. 2020; Pankajam et al. 2020; Dutta et al. 2017). Thus, the possibility of a 30% increase in the LOH event rate due to CCGD carriage is quite modest compared to that produced by natural variation. Similarly, an increase in the point mutation rate of 30% pales in comparison to the five-fold variation in mutation rates among several yeast strains in a single experiment (Pankajam et al. 2020) and the six-fold variation observed across the genome of a single yeast strain (Lang and Murray 2011).

While we did not observe significant changes in the genome-wide rates, we found a statistically significant difference in the distribution of LOH rates along Chromosome V in the CCGD-carrying strains. We also found evidence that the presence of Cas9 increases the proportion of interstitial LOH events relative to terminal LOH events. The biological reasons for these two effects remain unresolved. We were unable to identify any localized sequence features, such as similarity to the gRNA or enrichment of PAM sites, that would explain a shift in the distribution of LOH events on Chr V in the presence of the CCGD. It is therefore possible that the observed shift is simply a statistical aberration. Alternatively, if the shift is in fact a real biological effect, it could be driven by many weak off-target sites that we could not detect. It is also possible that we failed to find any relevant sequence features because we used a metric that did not predict the probabilities of genomic sequences to be targeted by the CRISPR-Cas9/gRNA complex with sufficient accuracy. Further analysis and experiments will be necessary to discriminate between these hypotheses.

Our observation that Cas9 apparently increases the proportion of interstitial LOH events without increasing the overall rate of LOH events is intriguing because it suggests that the Cas9 protein, rather than affecting the rate of dsDNA breaks, may instead alter which pathways the cell uses to repair the breaks. Although the molecular mechanisms for this effect are unclear, they could be connected to the fact that Cas9 binds to DNA for an extended time (Sternberg et al. 2014).

We propagated our yeast MA lines asexually, and the CCGD and the Cas9 gene were expressed constitutively. However, CCGDs are often designed to be expressed only transiently during meiosis. Given that the mechanisms of the activities of various DNA repair mechanisms differ between meiosis and mitosis (Mehta and Haber 2014), it is possible that the mutagenic effects of such CCGDs may be different from those reported here. However, we expect that the overall mutagenic effect would be even weaker than in our system simply because the genome in such systems would be less exposed to Cas9.

Overall, our main finding that a CCGD or the Cas9 alone induces weak and likely localized mutagenic effects suggests that the long-term evolutionary risks associated with CCGD carriage are probably acceptable. However, we emphasize that this work alone is not sufficient to accurately assess these risks. Indeed, our results were obtained in a unicellular model organism under lab conditions. It is a priori unclear how they will translate to multicellular organisms in natural environments. More importantly, in order to measure the effects of CCGDs or Cas9 on mutation rates, we purposefully eliminated all but the strongest selection pressures. However, even weak selection pressures imposed on the organism by the presence of a CCGD element may be sufficient to change its long-term evolutionary trajectory. Such selection pressures may be very hard to measure and even harder to predict a priori, since they may be highly variable depending on the payload gene, the target species and the environment. Thus, further investigations will be needed to further assess evolutionary risks of CCGDs. Our present results only reaffirm that CCGDs remain at this point a viable option for population control.

## Methods

### Experimental methods

#### Strain construction

We generated the W, C and D strains by mating two haploid yeast strains. One parent strain is derived from the laboratory *S. cerevisiae* strain BY4741 (BY; MATa his3Δ1 leu2Δ0 met15Δ0 ura3Δ0) and the other parent is derived from the vineyard strain YAN501 (RM; MAT_α_ his3Δ1 leu2Δ0 ura3Δ0 HO::KanMX). These two parent strains differ by ~40,000 SNPs (Brachman et al., 1998; Brem et al., 2002; Bloom et al., 2013). We replaced the his3Δ1 allele in BY parent with a functional version to facilitate diploid selection. We amplified the NatMX-GFP, NatMX-GFP-Cas9 and NatMX-GFP-Cas9-gRNA constructs from a previously constructed gene drive cassette (Dicarlo et al., 2015) and integrated each of them into each of the parent strains at the ADE2 locus (Chr XV 564476–566191) using standard yeast transformation methods. The gRNA sequence used here targets the 5’ end of the ADE2 gene, and the disruption of ADE2 produces an easily observed red colony phenotype (Dicarlo et al., 2015). Single colonies of BY and RM haploid parents containing each of the integrated constructs were mated, and correct diploid progeny were confirmed by PCR targeting the Ade2 region. Diploid founders were each derived from independently transformed and mated BY and RM parents to reduce the chance of multiple lines being established from a mutated founder.

#### Mutation accumulation experiment

For each of the three constructs, we used eight founders to establish 96 independent mutation accumulation lines, for a total of 285 MA lines. Each of these lines was independently propagated for 43 cycles of streaking to single colony isolation on YPD agar and incubating at 30°C for 48 h, for a total average of 800 generations. The colony closest to a pre-marked location on the plate was used to continue the next cycle to prevent any potential artificial selection effects. Founder and end-point clones were preserved by culturing single colonies in YPD liquid media to mid-log growth and freezing at −80°C in 15% glycerol.

#### Testing gene-drive activity

To test whether the gene drive elements remained active at the end of the mutation accumulation, we sampled six end-point D clones, three exhibiting low LOH accumulation and three chosen randomly. We sporulated and dissected tetrads for each diploid clone, and mated one of the resulting Mat-α haploids with two different Mat-a “tester” haploids derived from strain MJM64 (McDonald et al., 2016). One tester strain contained an intact target (IT) ADE2 sequence, while the other (No Target, NT) was engineered with 12 mismatches across the ADE2 target sequence to abolish gRNA targeting. D haploid genotypes were -ura and -KanMX, making them 5-Fluoroortic acid (5FOA) resistant and G418 susceptible. The tester strains had a URA locus under a haploid-specific promoter and had the KanMX locus, making them 5FOA susceptible as haploids and 5FOA and G418 resistant as diploids. We excluded tester haploids by selecting on 5FOA and Drive haploids by selecting on G418. Since the two selection components, 5FOA and G418, require different media pHs, we sequentially selected for diploids by first spot-plating diluted mating cultures on Complete Synthetic Media (CSM) +5FOA, then scraping and diluting each spot, and spot-plating on CSM +G418. After 48h of growth on CSM +G418, colonies were photographed and screened for Ade2 disruption by observing for red colonies.

#### Library preparation and sequencing

Genomic DNA was extracted and purified from each founder and end-point clone using a modified ethanol precipitation protocol. Briefly, overnight cultures were pelleted and resuspended in 6% SENT (6% SDS, 10mM EDTA, 30mM Tris, pH8) and incubated at 65°C for 15 min. Cooled lysates were combined with RNAse A and incubated at 37°C for 60 min. A half volume of 3M NaOAc was added and the mixture was centrifuged to pellet proteins and cellular components. The supernatant was washed first in isopropanol and then ice-cold ethanol. DNA pellets were air dried in inverted tubes and resuspended in nuclease-free water. We prepared whole-genome sequencing libraries using the Illumina Nextera system with a modified protocol (Baym et al. 2015). We pooled 100bp paired-end libraries and sequenced them on an Illumina Hi-Seq 4000 platform to a median high-quality read depth of 22x.

### Data analysis

#### Analysis of statistical power

We developed a Monte Carlo simulation method for determining the power of our MA experiment to detect differences in the rates of LOH events and point mutations. For a priori power estimates, we assumed that mutations would accumulate according to a Poisson process, and modeled mutation counts in a set of end-point clones as a set of random draws from a Poisson distribution. We calculated expected mutation count λ from previous rate estimates and our anticipated design of 95 end-point clones per treatment and 750 generations of propagation. For LOH events, this rate was estimated to be 2.5×10^−2^ per genome per generation (Sui et al. 2020; Pankajam et al. 2020), and so the expected number of LOH events in an end-point clone was 12. For point mutations, the estimated rate was 4.0×10^−3^ per genome per generation (Sui et al. 2020; Zhu et al. 2014), and so the expected number of point mutations was 3. We simulated our experiment by drawing 95 end-point clones from a Poisson distribution with λ equal to the expected number of mutations to form a “wild-type” sample. We generated a second “effect” sample of end-point clones from a Poisson distribution with λ greater than that of the “wild-type” by a particular effect size. We performed a permutation test of the difference in means between the “wild-type” and “effect” samples. This procedure of drawing two samples differing by an effect size and performing a permutation test was repeated for 1000 iterations. We calculated the power of our experimental setup as the fraction of significant permutation tests for each effect size. We chose specific sets of effect sizes for each mutation type that would capture the range of power in our experiment.

We calculated the empirical power curve for our experiment using a method similar to that of the a priori estimates, but rather than drawing a “wild-type” sample from a Poisson distribution, we generated the “wild-type” from the pooled mutation counts of all end-point clones in our experiment. This empirical distribution is not significantly different from Poisson, but is somewhat more dispersed. Thus, a Poisson distribution may overestimate the power of our experiment. Since we failed to detect a significant difference in mean LOH counts between the drive strains and the WT control, we pooled all end-point clones to form sampling population A. We then formed a second population (B) by adding LOH events to random clones such that the difference in means (effect size) between populations A and B varied between 10% and 100%. We then randomly sampled n clones from each population, with n equal to the smallest end-point clone sample size (66 in the Drive strain). We performed the same permutation test as in Fig 1 to obtain a p-value. We repeated this sampling and testing cycle for 10,000 iterations at each effect size to obtain a distribution of p-values and estimated the power as the proportion of p-values less than alpha = 0.05 and 0.01. A power curve was plotted and the effect size required for detection at a power threshold of 70% was estimated for each value of alpha.

#### Estimating the number of gene-drive deactivating mutations

Since our MA lines had been accumulating mutations for approximately 800 generations, it is possible that some mutations had hit and deactivated the gene drive element. Given that SNP and indels occur on average at rates of 1.67×10^–10^ and 7.5×10^–12^ per base pair per generation, respectively (Zhu et al. 2014; Pankajam et al. 2020; Lang and Murray 2008), and given that our constructs are 7999 bp, 7601 bp and 2770 bp long for the D, C, and W founders, respectively, we expect a typical end-point D, C and W clone to have 2.3×10^–3^, 2.2×10^–3^, and 0.8×10^–3^ mutations in the construct, respectively. Thus, we expect a total of 0.39 out of 223 sequenced end-point clones to carry a construct mutation. These estimates are based on the reasonable assumption that all mutations within the constructs are effectively neutral in our MA experiment, i.e., they do not provide a selective advantage or disadvantage massive enough to overcome the strong genetic drift imposed by periodic single-cell bottlenecks. A more important source of uncertainty in these estimates arise from the fact that they are based on genome-wide average mutation rate estimates, but the actual mutation rates can vary up to 6-fold across locations in the genome (Lang and Murray 2011). Thus, we might reasonably expect between 0.07 and 2.3 out of 223 sequenced clones to carry a mutation in the construct.

#### Read processing and variant calling

We trimmed reads using Trimmomatic 0.36 in the paired-end mode: Illumina adapter sequences and the leading and trailing 10 bases were removed, low quality end-regions were then removed (as determined by a sliding window of 6 bp and quality threshold of 10), and resulting reads of length less than 30 were removed.

We mapped the reads for each clone to the S288C-R64 reference sequence with bowtie2 with the --local-very-sensitive preset, allowing for gaps between paired reads of 100–1000bp, and retaining default values for all other settings. We removed duplicates with the default settings in Picard MarkDuplicates. We ran GATK HaplotypeCaller on each BAM alignment file with the flag -ERC GVCF, which outputs an intermediate gVCF file with genotype and likelihood values for every genomic position. GVCF files for all clones were pooled and final genotyping was performed with GATK GenotypeGVCFs. We filtered out low quality sites with GATK VariantFilteration and custom R code, removing calls with read depth < 6, mapping quality rank sum < −2.5, Fisher’s strand bias > 20.0, mapping quality < 50, and Phred-scaled site quality (QUAL) > 1000. Calls within repeat and low complexity regions, as identified using RepeatMasker, were also discarded.

To detect aneuploidies, we calculated the mean number of reads supporting the BY and RM alleles for each chromosome and for the whole genome across all clones. Chromosomes for which the mean coverage of one allele remained near the genome-wide average while the mean coverage for the other allele was near zero or at least 1.8x the genome-wide average were marked as aneuploids. There were no aneuploid events in the founders, but there were 18 events in 17 end-point clones. These aneuploid events were removed from LOH analysis. In order to detect whole-genome aneuploid events, we calculated the ratio of the mean BY allele coverage and the mean RM allele coverage for each chromosome. If all chromosomes in a clone had similar allele coverage ratios that were all near 0.5 or 2, it suggests that one set of parental chromosomes is present at twice the frequency of the other parental set. Two entire D strain founder groups (F_B and F_E) and one W strain end-point clone (N_B12) all had ratios near 0.5, indicating whole RM-genome amplifications, all of which were removed from LOH analysis.

High-throughput library preparation may produce instances of contamination between samples or poor sequencing output (Kinsler et al. 2023). If the library for one clone was contaminated with DNA from another clone, we may observe genomic regions with many homozygous calls, due to an LOH event in the ‘true’ sample, interspersed by sporadic heterozygous calls with high allele imbalance, due to the minor contribution of reads supporting the non-LOH allele. We found 13 end-point clones with this type of evidence for possible contamination, which were removed from all analyses. We obtained little or no sequencing coverage for two founders (H_F00 and H_H00). In order to rescue these MA lines for LOH and mutation analyses, we imputed the ancestral genotypes from descendant end-point clones. Following from the criteria for our erroneous call detection method described below, we imputed founder heterozygous calls by requiring at least four of the descendant end-point clones and four other founders to be called heterozygous and zero founders to be called homozygous. Homozygous calls were imputed by requiring at least seven end-point clones and four founders to have the same homozygous call, and allowing no founders and only one end-point clone to have a polymorphism. Inclusion of these imputed founders did not change the results we observed, but did allow for greater statistical power, finer spatial detail, and reduced sample size imbalance between treatments.

#### Dual-reference genotype reconciliation

We counted the fraction of reads supporting each allele at sites with heterozygous calls when aligned to the BY reference, and found a consistent bias toward the BY allele across clones (Supp. Fig. 5). After repeating read mapping against the RM reference, we found analogous reference-specific mapping bias, an issue also observed in previous work (Zheng and Emerson, 2019). In order to minimize the impact of this bias, we developed a method to reconcile the two callsets. We retained all calls that agreed between the two callsets so long as at least one of the GQ values was greater than 50. For calls that did not agree between the two references, we retained the call with the higher GQ value, so long as it exceeded an absolute threshold of 50 and exceeded the lower GQ value by at least 30. Discordant sites for which neither call had a GQ exceeding 50 or for which one call was missing were discarded. After this procedure, false homozygous call rates were significantly lower than in the call sets derived from only the BY or RM reference (P = 0.0095, P = 0.0019, respectively; T-test; Supp. Fig. 2).

To further increase our confidence in our callset given the mapping bias issue, we investigated the allele frequency distribution of heterozygous sites. We identified a set of true heterozygous sites as those in our LOH SNP set for which at least 7 founders were called heterozygous and none were homozygous. The total read depths of individual calls at these sites were sorted into 26 log-scaled read depth bins and the variance in reference allele frequency was calculated for each bin. The complement set of ‘unknown’ heterozygous calls was binned in the same fashion, and calls were removed if reference allele frequencies fell outside of the two-tailed 95% confidence intervals for their respective bins. This removed the 0.5% of heterozygous calls with the greatest imbalance in read allele frequency.

#### Estimation of genotyping error rates

In order to assess the rate of erroneous genotypes in our final callset, we developed a method for detecting them by exploiting the phylogenetic relationship of our clones. We reasoned that the same erroneous call arising in multiple end-point clones should be rare. Thus, if many (at least 4) end-point clones derived from the same founder had the same heterozygous call, this was assumed to be the true ancestral genotype at that site. If the founder of that set of end-point clones was homozygous at that site, we counted that as a false homozygous error. Similarly, if all end-point clones in a founder group had the same homozygous call, with the support of at least seven clones, but the founder was heterozygous, we counted this as a false heterozygous error. Sites for which a confident true ancestral call could not be obtained due to a mixture of calls likely represent polymorphisms that arose during strain construction, and so were excluded from error estimation.

#### LOH event detection

After processing and filtering genotype calls, we recovered on average 32,188 (range: 23,910 – 39,189) ancestral heterozygous SNP markers in each clone. Ancestral indel polymorphisms were excluded from the set of LOH markers, as they are called less reliably and represent only a small fraction of all ancestral polymorphisms. Due to differences in sequencing coverage and genotyping quality, each clone had an overlapping but unique set of markers with assigned genotypes. Even with this heterogeneity in sampled markers, there was strong overlap within founder groups, such that, on average, 91% of markers were present in all end-point clones descended from the same founder. Further, we did not observe a significant correlation between the number of markers sampled and the number of LOH events detected (Adj. R^2^ = −0.0040, P = 0.725, linear regression), which suggests that, within the range of markers we sampled, our ability to detect LOH events is not diminished in clones with fewer markers. We found that marker density was also heterogeneous among chromosomes, with lower density in larger chromosomes. However, we again did not observe a significant correlation between chromosomal marker density and LOH event rate (Adj. R^2^ = 0.029, P = 0.248, linear regression).

We observed 37 markers with numerous apparent LOH conversions, but nearly all converted to the same homolog. We reasoned that either these markers resided in regions both highly prone to mitotic recombination and with an extreme fitness difference between conversions to each homolog, or they are prone to sequencing error. Given the minimal selective regime of our experiment and the fact that these regions had not been identified as highly biased in previous studies using this hybrid, we reasoned that the latter is more likely. Therefore, if a marker had undergone more than 9 conversions to one homolog and at most one conversion to the alternative homolog, this marker was removed from LOH detection.

We detected LOH events by comparing the set of heterozygous markers in a founder against all end-point clones that descended from it. Contiguous markers that were converted to a particular allele in an end-point clone were merged into LOH tracts. The boundaries of each LOH tract were estimated as the midpoints between the first and last converted markers and their nearest unconverted markers. As observed previously (Lee et al. 2009; Yin and Petes 2013; Zheng et al. 2016; Sui et al. 2020), single dsDNA breaks can induce complex repair patterns that include regions of homozygosity from either or both homologous chromosomes and heterozygous regions. Thus, we merged LOH tracts that were on the same chromosome and with boundaries separated by less than 10 kbp into LOH events.

Given the false homozygous error rate estimated above (see “Estimation of genotyping error rates“) and the number of markers we sampled across all end-point clones, we calculated an expected number of erroneous homozygous calls. While we do not know which specific calls are erroneous, an LOH event supported by only one converted marker is more likely to be erroneous than one supported by multiple markers. The number of expected false homozygous calls (598) exceeded the number of single-marker LOH events (526). Therefore, we removed all singleton LOH events. The remaining expected false homozygous markers must be present in multi-marker LOH events. The same reasoning should apply that a two-marker LOH event is more likely to be erroneous than one supported by a greater number of markers. Therefore, we performed a Monte Carlo simulation by randomly sampling the remaining 69 expected erroneous markers from two-marker LOH events over 1000 iterations to obtain the expected number of erroneous two-marker LOH events. A mean of 8 out of 153 (5.2%) two-marker LOH events were expected to be erroneous and 52 were expected to contain one erroneous marker. Since there were so few expected errors, we opted to include all two-marker LOH events.

To observe for positional effects associated with LOH breakpoints in the strains, we first estimated the positions of breakpoints for interstitial and terminal LOH events separately. Since we cannot detect the exact location of the dsDNA break that initiated LOH formation, for interstitial LOH events, we located the breakpoint as the midpoint of the LOH boundaries estimated above (Hum and Jinks-Robertson 2017). Break induced replication (BIR) repair, which produces terminal LOH events, can result in DNA resection, and thus LOH extension, up to 69kb upstream (centromere proximal) from the break event, though it is unclear how this varies with position or other factors (Malkova et al. 2001). Therefore, similar to a previous study, we defined breakpoint positions of terminal LOH events to be 10kb from the centromere-proximal boundary estimated above (Sui et al. 2020).

We analyzed whether LOH breakpoint and conversion rates differed among the strains at three levels of resolution: genome-wide, by chromosome, and along 50kb sliding windows. Since chromosomes do not divide evenly by the window size, we held window size fixed, while reducing the shift distance such that the first and last windows fit within the chromosome end positions. For the genome-wide rate estimates of each strain, we calculated the LOH breakpoint rate as the per-genome per-generation rate by dividing the total number of LOH events by the number of clones and number of generations. For finer resolutions, we calculated the per-base-pair per generation rate by dividing the number of events in a window (chromosome or 50kb region) by the length of the window and the number of generations.

We tested whether the positional distribution of all LOH breakpoints along each chromosome was heterogeneous by comparing the empirical cumulative distribution (ECD) with that of a uniform distribution. For this, we used the Hegazy goodness-of-fit test, which calculates the mean distance between two cumulative distributions (Hegazy and Green 1975). P-values were calculated from 2000 iterations of a Monte Carlo simulation sampling from a uniform distribution. To test whether two samples had come from the same positional distribution, we used the Wasserstein statistic, which is calculated as the integral of the function |E(x) - F(x)|, where E and F are the ECDFs of the two samples (Vaserstein 1969). P-values were calculated from 2000 iterations of a Monte Carlo simulation sampling from the joint distribution of the two samples.

We also investigated whether the LOH conversion rates were different among strains at the same three levels of resolution. Genome-wide rates were calculated as the sum of estimated LOH event lengths across clones in a strain by the number of clones, number of generations, and length of the diploid genome. Chromosomal rates were estimated in the same way, but using the length of each chromosome multiplied by 2 for diploidy. Sliding windows were constructed as described above, but because LOH events can span many windows and span partial windows, the rate calculation differed from that of breakpoints. Specifically, for each window and clone, we calculated the proportion of that window that had been converted by an LOH event, given the boundaries estimated above. We then took the mean proportion of the window converted across clones in a strain and divided this quantity by the number of generations and size of the window to get a per-base-pair per-generation rate.

Another consequence of the extended nature of LOH events is that, unlike breakpoints, we cannot use the ECD to estimate differences in the positional distributions. In order to balance the desire for fine scale resolution and the negative relationship between number of statistical tests and statistical power, we opted to estimate differences in position-specific rates at the level of one-third chromosome. This works well, as it limits the number of comparisons, but also captures much of the variability, which is primarily located at the ends of chromosomes. Therefore, we split each chromosome into three, equally sized windows and applied the same rate estimate procedure as that for the 50kb windows. We then tested for significant differences with a permutation test and Benjamini-Hochberg correction.

#### Detection of de novo mutations

The complementary set of polymorphisms to the set of LOH markers represents potential de-novo mutations. However, among these putative mutations, we observed many sites where multiple end-point clones possessed the same polymorphism. Given that the mutation rates in yeast are estimated to be about 1.67×10^–10^ (Zhu et al. 2014), the probability for the same mutation to arise independently in two or more clones in our entire MA experiment is 1.3×10^–3^. Therefore, we identified those variants that were present in only a single end-point clone as new mutations. While this approach is expected to remove many spurious variant calls, as was the case for LOH markers, we observed that some heterozygous calls exhibited a strong bias in the fraction of reads supporting each allele away from the expected 50%. Therefore, we used the same heterozygous call filtering method described above and removed 0.6% of all variant calls that represent likely errors. In 25 cases, apparent complex mutation events had occurred, wherein two or more mutations were present within 100bp of each other, and so we merged these variant calls into one mutation event. We subjected the triploid MA lines to this same pipeline and found similar rates of point mutations, thus they were included in the following analyses.

Point mutation distributions along the genome were generally uniform. Among chromosomes, the highest and lowest per base-pair per generation rates differed by only by 1.7x, and the inclusion of gene drive elements do not appear to have a significant effect on the rate distribution among chromosomes (P = 0.242, P = 0.265, χ^2^ test). At the sub-chromosomal level, mutation rates tended to fluctuate more dramatically. However, after multiple-testing correction, these deviations from uniformity were not significant (P > 0.167, Hegazy uniformity test). Furthermore, when comparing the D and C strains against the W strain, we did not detect any significant deviations in the positional distributions of mutations for any chromosome (Adjusted P > 0.92, Wasserstein test). While we could not reject the hypothesis that this heterogeneity in mutation rates were due to random fluctuations, they could still be the result of low level Cas9 activity. Therefore, we tested whether these peaks contain significantly higher gRNA similarity or PAM site density (described below). Regions with elevated mutation rates in the C- and D-strains did not have significantly greater mean or maximum gRNA similarity than elsewhere in the genome (C strain: P = 0.11, D strain: P = 0.256, permutation tests). Similarly, we did not observe any significant increases in PAM site density in regions with high mutation rates (C strain: P = 0.26, D strain P = 0.062, permutation tests). These results provide further support that the observed rate differences in the D and C strains are random in nature, and not driven by off-target Cas9 activity..

#### gRNA similarity scoring

In order to detect genomic regions of complementarity with the gRNA, we generated a scoring system based on the empirical changes in Cas9 activity given single and multiple mismatches at all positions (Hsu et al., 2013). Since the effect of a mismatch is dependent on its position relative to the PAM, we generated a linear model relating mismatch position and Cas9 activity reduction. Multiple mismatches can also further reduce Cas9 activity, and so, in addition to a position-specific penalty for each mismatch, a run length penalty was also applied. The Cas9-gRNA complex first interrogates the genome for the presence of PAM sites, and a PAM sequence is required for Cas9 nuclease activity, so we first indexed the positions of all -NGG PAM sequences, as well as the alternative form -NAG, on both strands of the S288C_R64 reference genome (Sternberg et al., 2014; Leenay et al., 2016). For each PAM position, we scored the 20-bp region immediately upstream against our target gRNA sequence.

We looked for additional evidence that would support such CCGD off-target activity on Chr V. The Cas9-gRNA complex cuts dsDNA at those sites in the genome that are adjacent to PAM sites and whose sequence is close to complementary to the sequence of the gRNA (Sternberg et al. 2014). Across both the BY and RM reference sequences, we uniquely identified a total of 801,751 -NGG PAM sites and 1,4093,38 less favorable -NAG PAM sites in the yeast genome. Of these, 6192 -NGG and 10,542 -NAG were between positions 180kb and 271kb on Chr V, that is, close to the apparent LOH hotspot found in the D clones. We evaluated how similar the 20 bp sequences 5’-adjacent to these PAM sites are to the complement of our gRNA (Methods). We found that PAM-adjacent sequences near the LOH hotspot on Chr V are no more similar to the gRNA sequence complement than PAM-adjacent sequences elsewhere in the genome, whether we test the mean or maximum similarity score (*P* = 0.92, P = 0.78, respectively; permutation test). We repeated this procedure for the other D hotspot on Chr II and also found no significant increase in gRNA similarity (*P* = 0.16, P = 0.23, respectively; permutation test). Previous work has shown that apo-Cas9, that is the Cas9 protein unassociated with a gRNA, can strongly interact with DNA at PAM sites, and so we tested whether the hotspots identified for the C strain contained a greater density of PAM sites (Sternberg et al. 2014). Specifically, we pooled all 50kb windows from above that contained greater than five breakpoints in the C strain and tested whether the number of PAM sites was significantly greater in this pool than the rest of the genome. We found no such enrichment for PAM sites at C strain hotspots (P = 0.72, permutation test).

## Supplementary Material

1

## Figures and Tables

**Table 1.** Key mutation counts and rates among end-point clones from the three strains.

Strain	Loss of Heterozygosity					Point mutations			Indels	
	End-point clones	Total Events	Event rate (×10^−10^)^1^	RM/BY to BY/BY	Total interstitial	End-point clones	Total	Rate (×10^−10^)^1^	Total	Rate (×10^−11^)^1^
W	75	376	2.99 ± 0.37	183 (48.7%)	235 (62.5%)	75	418	3.32 ± 0.33	84	6.67 ± 1.59
C	84	456	3.23 ± 0.31	247 (54.2%)	328 (71.9%)	84	469	3.33 ± 0.39	86	6.10 ± 1.56
D	64	322	3.00 ± 0.34	164 (50.9%)	203 (63%)	89	435	2.91 ± 0.31	83	5.55 ± 1.07

1 per base-pair per generation ± 95% bootstrap confidence interval

## References

[R1] Adolfi Adriana, Gantz Valentino M., Jasinskiene Nijole, Lee Hsu-Feng, Hwang Kristy, Terradas Gerard, Bulger Emily A., 2020. “Efficient Population Modification Gene-Drive Rescue System in the Malaria Mosquito Anopheles Stephensi.” Nature Communications 11 (1): 5553. 10.1038/s41467-020-19426-0 PMC7609566 33144570

[R2] Baym Michael, Kryazhimskiy Sergey, Lieberman Tami D., Chung Hattie, Desai Michael M., and Kishony Roy. 2015. “Inexpensive Multiplexed Library Preparation for Megabase-Sized Genomes.” PloS One 10 (5): e0128036. 26000737 10.1371/journal.pone.0128036 PMC4441430

[R3] Beaghton Andrea K., Hammond Andrew, Nolan Tony, Crisanti Andrea, and Burt Austin. 2019. “Gene Drive for Population Genetic Control: Non-Functional Resistance and Parental Effects.” Proceedings. Biological Sciences / The Royal Society 286 (1914): 20191586. 10.1098/rspb.2019.1586 PMC6842858 31662083

[R4] Bier Ethan. 2021. “Gene Drives Gaining Speed.” Nature Reviews. Genetics 23 (1): 5–22. 10.1038/s41576-021-00386-0 PMC8344398 34363067

[R5] Bloom Joshua S., Ehrenreich Ian M., Loo Wesley T., Lan Võ Lite Thúy, and Kruglyak Leonid. 2013. “Finding the Sources of Missing Heritability in a Yeast Cross.” Nature 494 (7436): 234–37. 23376951 10.1038/nature11867 PMC4001867

[R6] Boyle Evan A., Becker Winston R., Bai Hua B., Chen Janice S., Doudna Jennifer A., and Greenleaf William J.. 2021. “Quantification of Cas9 Binding and Cleavage across Diverse Guide Sequences Maps Landscapes of Target Engagement.” Science Advances 7 (8). 10.1126/sciadv.abe5496. PMC7895440 33608277

[R7] Champer Jackson, Buchman Anna, and Akbari Omar S.. 2016. “Cheating Evolution: Engineering Gene Drives to Manipulate the Fate of Wild Populations.” Nature Reviews. Genetics 17 (3): 146–59. 10.1038/nrg.2015.34 26875679

[R8] Champer Jackson, Reeves Riona, Oh Suh Yeon, Liu Chen, Liu Jingxian, Clark Andrew G., and Messer Philipp W.. 2017. “Novel CRISPR/Cas9 Gene Drive Constructs Reveal Insights into Mechanisms of Resistance Allele Formation and Drive Efficiency in Genetically Diverse Populations.” PLoS Genetics 13 (7): e1006796. 28727785 10.1371/journal.pgen.1006796 PMC5518997

[R9] Cradick Thomas J., Fine Eli J., Antico Christopher J., and Bao Gang. 2013. “CRISPR/Cas9 Systems Targeting β-Globin and CCR5 Genes Have Substantial off-Target Activity.” Nucleic Acids Research 41 (20): 9584–92. 23939622 10.1093/nar/gkt714 PMC3814385

[R10] DiCarlo James E., Chavez Alejandro, Dietz Sven L., Esvelt Kevin M., and Church George M.. 2015. “Safeguarding CRISPR-Cas9 Gene Drives in Yeast.” Nature Biotechnology 33 (12): 1250–55. 10.1038/nbt.3412 PMC4675690 26571100

[R11] Dutta Abhishek, Dutreux Fabien, and Schacherer Joseph. 2021. “Loss of Heterozygosity Results in Rapid but Variable Genome Homogenization across Yeast Genetic Backgrounds.” eLife 10 1–19. 10.7554/eLife.70339 PMC8245132 34159898

[R12] Dutta Abhishek, Lin Gen, Pankajam Ajith V., Chakraborty Parijat, Bhat Nahush, Steinmetz Lars M., and Nishant Koodali T.. 2017. “Genome Dynamics of Hybrid Saccharomyces Cerevisiae During Vegetative and Meiotic Divisions.” G3 7 (11): 3669–79. 28916648 10.1534/g3.117.1135 PMC5677154

[R13] Esvelt Kevin M., Smidler Andrea L., Catteruccia Flaminia, and Church George M.. 2014. “Concerning RNA-Guided Gene Drives for the Alteration of Wild Populations.” eLife 3 (July). 10.7554/eLife.03401. PMC4117217 25035423

[R14] Frankham R. 1995. “CONSERVATION GENETICS.” Annual Review of Genetics 29 (1): 305–27. 10.1146/annurev.ge.29.120195.001513 8825477

[R15] Fu Yanfang, Foden Jennifer A., Khayter Cyd, Maeder Morgan L., Reyon Deepak, Joung J. Keith, and Sander Jeffry D.. 2013. “High-Frequency off-Target Mutagenesis Induced by CRISPR-Cas Nucleases in Human Cells.” Nature Biotechnology 31 (9): 822–26. 10.1038/nbt.2623 PMC3773023 23792628

[R16] Gantz Valentino M., and Bier Ethan. 2015. “Genome Editing. The Mutagenic Chain Reaction: A Method for Converting Heterozygous to Homozygous Mutations.” Science 348 (6233): 442–44. 25908821 10.1126/science.aaa5945 PMC4687737

[R17] Gantz Valentino M., Jasinskiene Nijole, Tatarenkova Olga, Fazekas Aniko, Macias Vanessa M., Bier Ethan, and James Anthony A.. 2015. “Highly Efficient Cas9-Mediated Gene Drive for Population Modification of the Malaria Vector Mosquito Anopheles Stephensi.” Proceedings of the National Academy of Sciences of the United States of America 112 (49): E6736–43. 26598698 10.1073/pnas.1521077112 PMC4679060

[R18] Gou Liangke, Bloom Joshua S., and Kruglyak Leonid. 2019. “The Genetic Basis of Mutation Rate Variation in Yeast.” Genetics 211 (2): 731–40. 30504363 10.1534/genetics.118.301609 PMC6366923

[R19] Hegazy Y. A. S., and Green J. R.. 1975. “Some New Goodness-of-Fit Tests Using Order Statistics.” Journal of the Royal Statistical Society. Series C, Applied Statistics 24 (3): 299–308.

[R20] Hsu Patrick D., Scott David A., Weinstein Joshua A., Ran F. Ann, Konermann Silvana, Agarwala Vineeta, Li Yinqing, 2013. “DNA Targeting Specificity of RNA-Guided Cas9 Nucleases.” Nature Biotechnology 31 (9): 827–32. 10.1038/nbt.2647 PMC3969858 23873081

[R21] Hum Yee Fang, and Jinks-Robertson Sue. 2017. “Mitotic Gene Conversion Tracts Associated with Repair of a Defined Double-Strand Break in Saccharomyces Cerevisiae.” bioRxiv. 10.1101/132167. PMC5586366 28743762

[R22] James Timothy Y., Michelotti Lucas A., Glasco Alexander D., Clemons Rebecca A., Powers Robert A., James Ellen S., Simmons D. Rabern, Bai Fengyan, and Ge Shuhua. 2019. “Adaptation by Loss of Heterozygosity in Saccharomyces Cerevisiae Clones Under Divergent Selection.” Genetics 213 (2): 665–83. 31371407 10.1534/genetics.119.302411 PMC6781901

[R23] Kandul Nikolay P., Liu Junru, Bennett Jared B., Marshall John M., and Akbari Omar S.. 2021. “A Confinable Home-and-Rescue Gene Drive for Population Modification.” eLife 10 (March). 10.7554/eLife.65939. PMC7968924 33666174

[R24] Kinsler Grant, Schmidlin Kara, Newell Daphne, Eder Rachel, Apodaca Sam, Lam Grace, Petrov Dmitri, and Geiler-Samerotte Kerry. 2023. “Extreme Sensitivity of Fitness to Environmental Conditions: Lessons from #1BigBatch.” Journal of Molecular Evolution 91 (3): 293–310. 37237236 10.1007/s00239-023-10114-3 PMC10276131

[R25] Labib Karim, and Hodgson Ben. 2007. “Replication Fork Barriers: Pausing for a Break or Stalling for Time?” EMBO Reports 8 (4): 346–53. 17401409 10.1038/sj.embor.7400940 PMC1852754

[R26] Lang Gregory I., and Murray Andrew W.. 2008. “Estimating the per-Base-Pair Mutation Rate in the Yeast Saccharomyces Cerevisiae.” Genetics 178 (1): 67–82. 18202359 10.1534/genetics.107.071506 PMC2206112

[R27] ———. 2011. “Mutation Rates across Budding Yeast Chromosome VI Are Correlated with Replication Timing.” Genome Biology and Evolution 3 (June): 799–811. 21666225 10.1093/gbe/evr054 PMC3170098

[R28] Lee Phoebe S., Greenwell Patricia W., Dominska Margaret, Gawel Malgorzata, Hamilton Monica, and Petes Thomas D.. 2009. “A Fine-Structure Map of Spontaneous Mitotic Crossovers in the Yeast Saccharomyces Cerevisiae.” PLoS Genetics 5 (3). 10.1371/journal.pgen.1000410. PMC2646836 19282969

[R29] Li Bei, Wei Aiying, Song Chunxia, Li Ning, and Zhang Juren. 2008. “Heterologous Expression of the TsVP Gene Improves the Drought Resistance of Maize.” Plant Biotechnology Journal 6 (2): 146–59. 17999658 10.1111/j.1467-7652.2007.00301.x

[R30] Lin Yanni, Cradick Thomas J., Brown Matthew T., Deshmukh Harshavardhan, Ranjan Piyush, Sarode Neha, Wile Brian M., Vertino Paula M., Stewart Frank J., and Bao Gang. 2014. “CRISPR/Cas9 Systems Have off-Target Activity with Insertions or Deletions between Target DNA and Guide RNA Sequences.” Nucleic Acids Research 42 (11): 7473–85. 24838573 10.1093/nar/gku402 PMC4066799

[R31] Liu Haoxuan, and Zhang Jianzhi. 2019. “Yeast Spontaneous Mutation Rate and Spectrum Vary with Environment.” Current Biology: CB 29 (10): 1584–91.e3. 31056389 10.1016/j.cub.2019.03.054 PMC6529271

[R32] Loeillet Sophie, Herzog Mareike, Puddu Fabio, Legoix Patricia, Baulande Sylvain, Jackson Stephen P., and Nicolas Alain G.. 2020. “Trajectory and Uniqueness of Mutational Signatures in Yeast Mutators.” Proceedings of the National Academy of Sciences of the United States of America 117 (40): 24947–56. 32968016 10.1073/pnas.2011332117 PMC7547211

[R33] Malkova A., Ivanov E. L., and Haber J. E.. 1996. “Double-Strand Break Repair in the Absence of RAD51 in Yeast: A Possible Role for Break-Induced DNA Replication.” Proceedings of the National Academy of Sciences of the United States of America 93 (14): 7131–36. 8692957 10.1073/pnas.93.14.7131 PMC38948

[R34] Malkova A., Signon L., Schaefer C. B., Naylor M. L., Theis J. F., Newlon C. S., and Haber J. E.. 2001. “RAD51-Independent Break-Induced Replication to Repair a Broken Chromosome Depends on a Distant Enhancer Site.” Genes & Development 15 (9): 1055–60. 11331601 10.1101/gad.875901 PMC312680

[R35] Mandegar Mohammad A., and Otto Sarah P.. 2007. “Mitotic Recombination Counteracts the Benefits of Genetic Segregation.” Proceedings of the Royal Society B: Biological Sciences 274 (1615): 1301–7. 10.1098/rspb.2007.0056 PMC2176173 17360283

[R36] Marshall John M., and Akbari Omar S.. 2018. “Can CRISPR-Based Gene Drive Be Confined in the Wild? A Question for Molecular and Population Biology.” ACS Chemical Biology 13 (2): 424–30. 29370514 10.1021/acschembio.7b00923

[R37] Marshall John M., Buchman Anna, Sánchez C Héctor M., and Akbari Omar S.. 2017. “Overcoming Evolved Resistance to Population-Suppressing Homing-Based Gene Drives.” Scientific Reports 7 (1): 3776. 28630470 10.1038/s41598-017-02744-7 PMC5476637

[R38] Mehta Anuja, and Haber James E.. 2014. “Sources of DNA Double-Strand Breaks and Models of Recombinational DNA Repair.” Cold Spring Harbor Perspectives in Biology 6 (9): 1–17. 10.1101/cshperspect.a016428 PMC4142968 25104768

[R39] Newton Matthew D., Taylor Benjamin J., Driessen Rosalie P. C., Roos Leonie, Cvetesic Nevena, Allyjaun Shenaz, Lenhard Boris, Cuomo Maria Emanuela, and Rueda David S.. 2019. “DNA Stretching Induces Cas9 off-Target Activity.” Nature Structural & Molecular Biology 26 (3): 185–92. 10.1038/s41594-019-0188-z PMC7613072 30804513

[R40] Oberhofer Georg, Ivy Tobin, and Hay Bruce A.. 2018. “Behavior of Homing Endonuclease Gene Drives Targeting Genes Required for Viability or Female Fertility with Multiplexed Guide RNAs.” Proceedings of the National Academy of Sciences of the United States of America 115 (40): E9343–52. 30224454 10.1073/pnas.1805278115 PMC6176634

[R41] Pankajam Ajith V., Dash Suman, Saifudeen Asma, Dutta Abhishek, and Nishant Koodali T.. 2020. “Loss of Heterozygosity and Base Mutation Rates Vary among Saccharomyces Cerevisiae Hybrid Strains.” G3 Genes, Genomes, Genetics 10 (9): 3309–19. 32727920 10.1534/g3.120.401551 PMC7466981

[R42] Pattanayak Vikram, Lin Steven, Guilinger John P., Ma Enbo, Doudna Jennifer A., and Liu David R.. 2013. “High-Throughput Profiling of off-Target DNA Cleavage Reveals RNA-Programmed Cas9 Nuclease Specificity.” Nature Biotechnology 31 (9): 839–43. 10.1038/nbt.2673 PMC3782611 23934178

[R43] Rode Nicolas O., Estoup Arnaud, Bourguet Denis, Courtier-Orgogozo Virginie, and Débarre Florence. 2019. “Population Management Using Gene Drive: Molecular Design, Models of Spread Dynamics and Assessment of Ecological Risks.” Conservation Genetics 20 (4): 671–90.

[R44] Rosen Danielle M., Younkin Ellen M., Miller Shaylynn D., and Casper Anne M.. 2013. “Fragile Site Instability in Saccharomyces Cerevisiae Causes Loss of Heterozygosity by Mitotic Crossovers and Break-Induced Replication.” PLoS Genetics 9 (9): e1003817. 24068975 10.1371/journal.pgen.1003817 PMC3778018

[R45] Shyian Maksym, and Shore David. 2021. “Approaching Protein Barriers: Emerging Mechanisms of Replication Pausing in Eukaryotes.” Frontiers in Cell and Developmental Biology 9 (May): 672510. 34124054 10.3389/fcell.2021.672510 PMC8194067

[R46] Smukowski Heil Caiti S., DeSevo Christopher G., Pai Dave A., Tucker Cheryl M., Hoang Margaret L., and Dunham Maitreya J.. 2017. “Loss of Heterozygosity Drives Adaptation in Hybrid Yeast.” Molecular Biology and Evolution 34 (7): 1596–1612. 28369610 10.1093/molbev/msx098 PMC5455960

[R47] Song Wei, Dominska Margaret, Greenwell Patricia W., and Petes Thomas D.. 2014. “Genome-Wide High-Resolution Mapping of Chromosome Fragile Sites in Saccharomyces Cerevisiae.” Proceedings of the National Academy of Sciences of the United States of America 111 (21): E2210–18. 24799712 10.1073/pnas.1406847111 PMC4040566

[R48] Sternberg Samuel H., Redding Sy, Jinek Martin, Greene Eric C., and Doudna Jennifer A.. 2014. “DNA Interrogation by the CRISPR RNA-Guided Endonuclease Cas9.” Nature 507 (7490): 62–67. 24476820 10.1038/nature13011 PMC4106473

[R49] Sui Yang, Qi Lei, Wu Jian Kun, Wen Xue Ping, Tang Xing Xing, Ma Zhong Jun, Wu Xue Chang, 2020. “Genome-Wide Mapping of Spontaneous Genetic Alterations in Diploid Yeast Cells.” Proceedings of the National Academy of Sciences of the United States of America 117 (45): 28191–200. 33106417 10.1073/pnas.2018633117 PMC7668089

[R50] Tsai Shengdar Q., Nguyen Nhu T., Malagon-Lopez Jose, Topkar Ved V., Aryee Martin J., and Joung J. Keith. 2017. “CIRCLE-Seq: A Highly Sensitive in Vitro Screen for Genome-Wide CRISPR-Cas9 Nuclease off-Targets.” Nature Methods 14 (6): 607–14. 28459458 10.1038/nmeth.4278 PMC5924695

[R51] Unckless Robert L., Clark Andrew G., and Messer Philipp W.. 2017. “Evolution of Resistance against CRISPR/Cas9 Gene Drive.” Genetics 205 (2): 827–41. 27941126 10.1534/genetics.116.197285 PMC5289854

[R52] Vaserstein Leonid Nisonovich. 1969. “Markov Processes over Denumerable Products of Spaces, Describing Large Systems of Automata.” Rossiiskaya Akademiya Nauk. Problemy Peredachi Informatsii 5 (3): 64–72.

[R53] Wang Sibao, and Jacobs-Lorena Marcelo. 2013. “Genetic Approaches to Interfere with Malaria Transmission by Vector Mosquitoes.” Trends in Biotechnology 31 (3): 185–93. 23395485 10.1016/j.tibtech.2013.01.001 PMC3593784

[R54] Wernberg Thomas, Coleman Melinda A., Bennett Scott, Thomsen Mads S., Tuya Fernando, and Kelaher Brendan P.. 2018. “Genetic Diversity and Kelp Forest Vulnerability to Climatic Stress.” Scientific Reports 8 (1): 1851. 29382916 10.1038/s41598-018-20009-9 PMC5790012

[R55] Yin Yi, and Petes Thomas D.. 2013. “Genome-Wide High-Resolution Mapping of UV-Induced Mitotic Recombination Events in Saccharomyces Cerevisiae.” PLoS Genetics 9 (10). 10.1371/journal.pgen.1003894. PMC3814309 24204306

[R56] Zheng Dao Qiong, Zhang Ke, Wu Xue Chang, Mieczkowski Piotr A., and Petes Thomas D.. 2016. “Global Analysis of Genomic Instability Caused by DNA Replication Stress in Saccharomyces Cerevisiae.” Proceedings of the National Academy of Sciences of the United States of America 113 (50): E8114–21. 27911848 10.1073/pnas.1618129113 PMC5167146

[R57] Zhu Yuan O., Siegal Mark L., Hall David W., and Petrov Dmitri A.. 2014. “Precise Estimates of Mutation Rate and Spectrum in Yeast.” Proceedings of the National Academy of Sciences of the United States of America 111 (22). 10.1073/pnas.1323011111. PMC4050626 24847077

