# Supplementary material for: Experimental Test of Evolutionary Safety of a CRISPR-Cas9 Gene-Drive Element
Source: bioRxiv. 2025 Jul 7:2023.11.28.569142. Originally published 2023 Nov 29. Preprint. [Version 2] doi: 10.1101/2023.11.28.569142 (PMC10705488; doi:10.1101/2023.11.28.569142)
Supplement: 1 [file NIHPP2023.11.28.569142V2-supplement-1.pdf]

bioRxiv preprint doi: https://doi.org/10.1101/2023.11.28.569142; this version posted July 7, 2025. The copyright holder for this preprint (which was not certified by peer review) is the author/funder, who has granted bioRxiv a license to display the preprint in perpetuity. It is made available under aCC-BY 4.0 International license.

Supplemental Figures

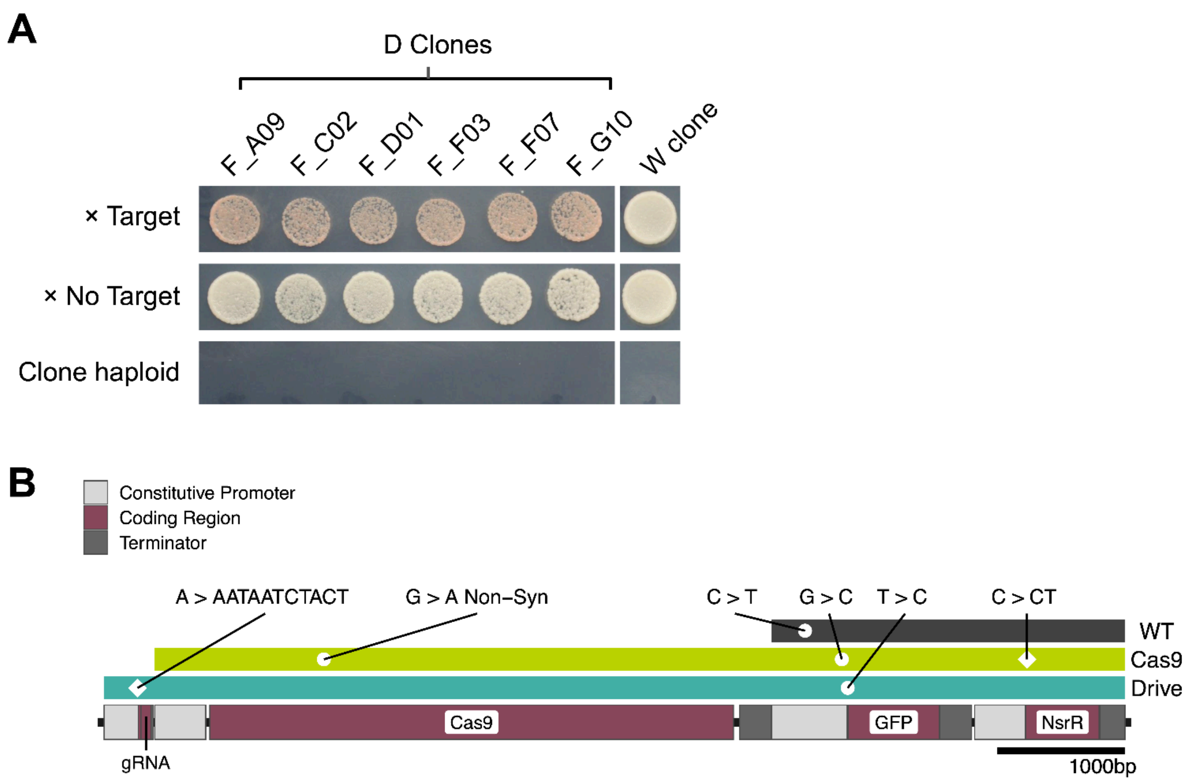

Figure S1. CCGD remains active throughout the MA experiment. A. All tested D end-point clones retain drive activity (see Section "Testing of gene-drive activity" in Materials and Methods). A haploid offspring of each tested D clone (columns) was mated either with a tester strain with the sequence targeted by the gRNA (Target) or with a tester strain without the target sequence (No target). Mated and unmated ("Clone haploid") liquid cultures were spot plated on CSM + 5FOA agar, then replica plated on CSM + G418 agar for diploid selection and incubated for 48 hours. A successful disruption of the ADE2 gene by the CCGD results in red colony coloration[33]. **B.** Mutations found in the construct. The construct map shows the position of each component and the three colored bars above represent the portion of the construct integrated into each strain. Circles indicate point mutations and diamonds indicate indels.

bioRxiv preprint doi: https://doi.org/10.1101/2023.11.28.569142; this version posted July 7, 2025. The copyright holder for this preprint (which was not certified by peer review) is the author/funder, who has granted bioRxiv a license to display the preprint in perpetuity. It is made available under aCC-BY 4.0 International license.

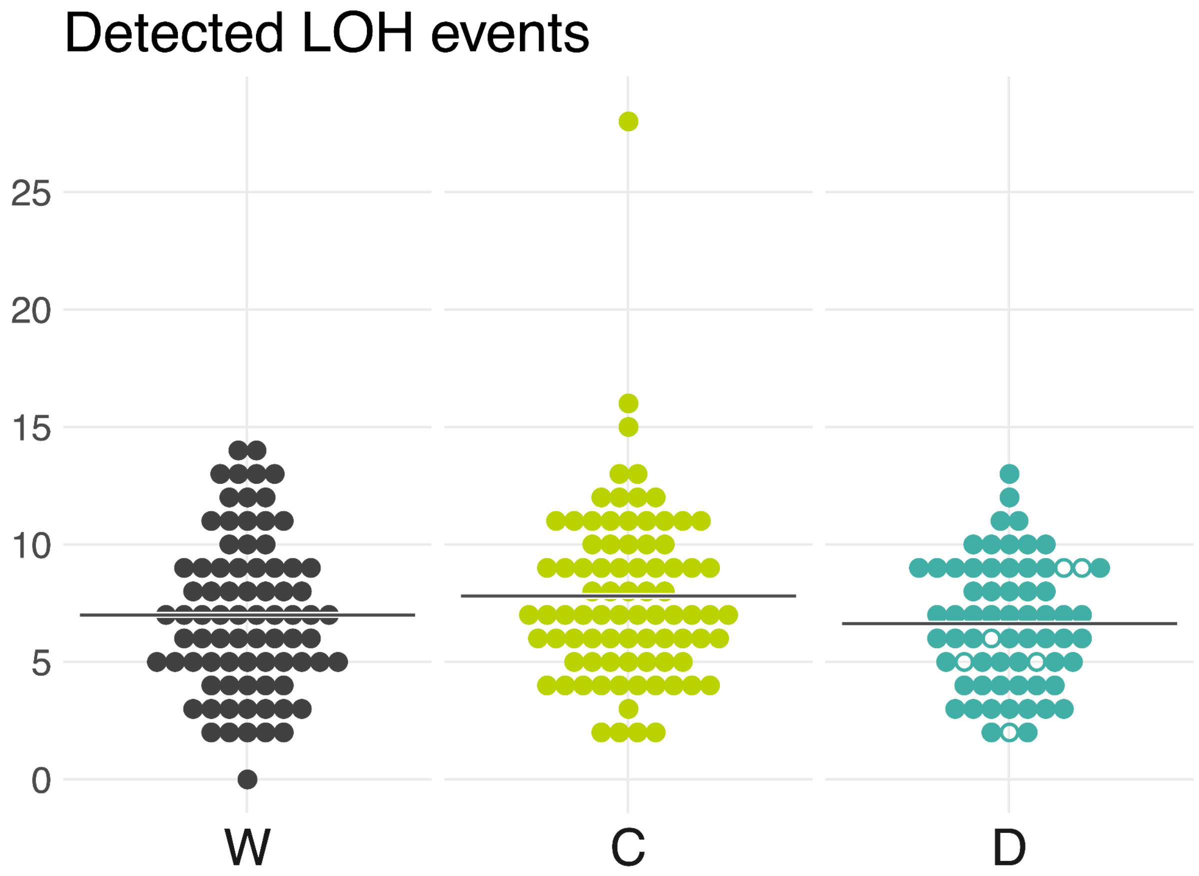

Figure S2. Distributions of detected LOH events across MA lines. Same as Figure 2B, but prior to undetected event corrections.

bioRxiv preprint doi: https://doi.org/10.1101/2023.11.28.569142; this version posted July 7, 2025. The copyright holder for this preprint (which was not certified by peer review) is the author/funder, who has granted bioRxiv a license to display the preprint in perpetuity. It is made available under aCC-BY 4.0 International license.

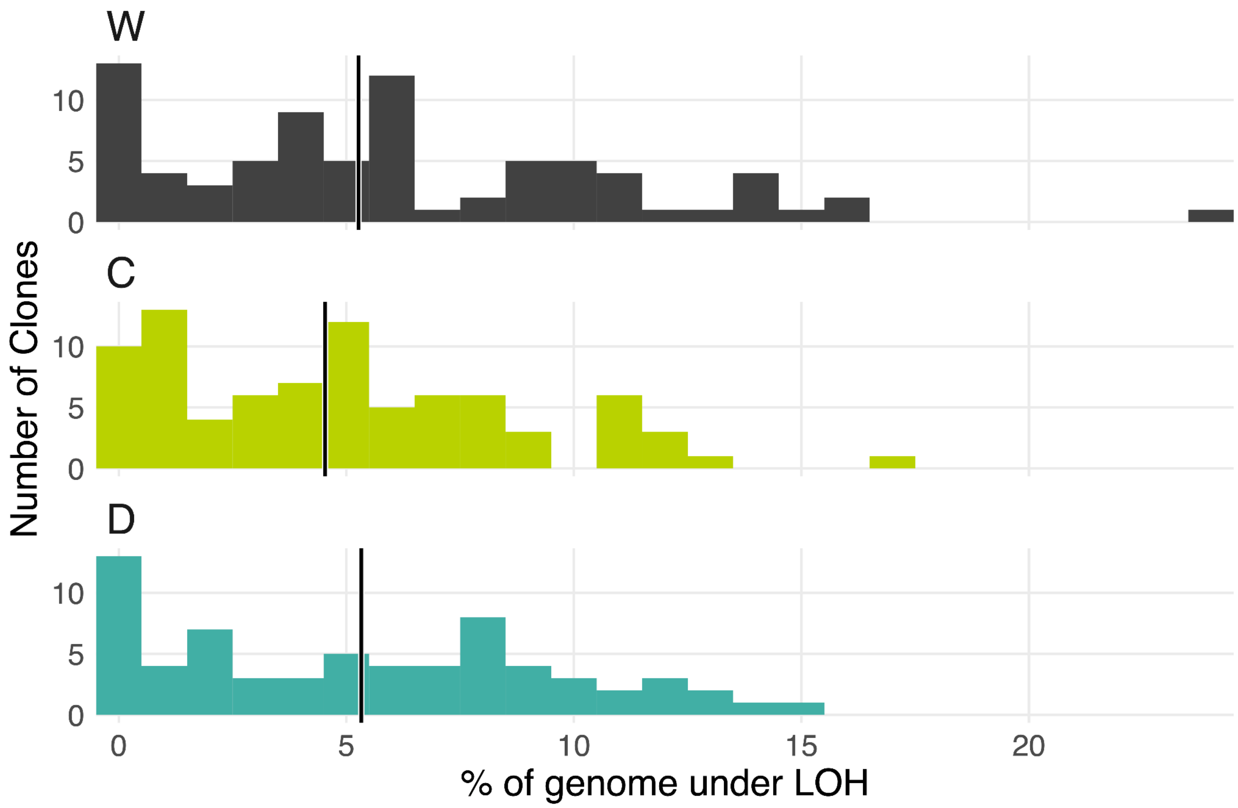

Figure S3. The rate of LOH conversion is not statistically elevated in CCGD element carrying strains.

bioRxiv preprint doi: https://doi.org/10.1101/2023.11.28.569142; this version posted July 7, 2025. The copyright holder for this preprint (which was not certified by peer review) is the author/funder, who has granted bioRxiv a license to display the preprint in perpetuity. It is made available under aCC-BY 4.0 International license.

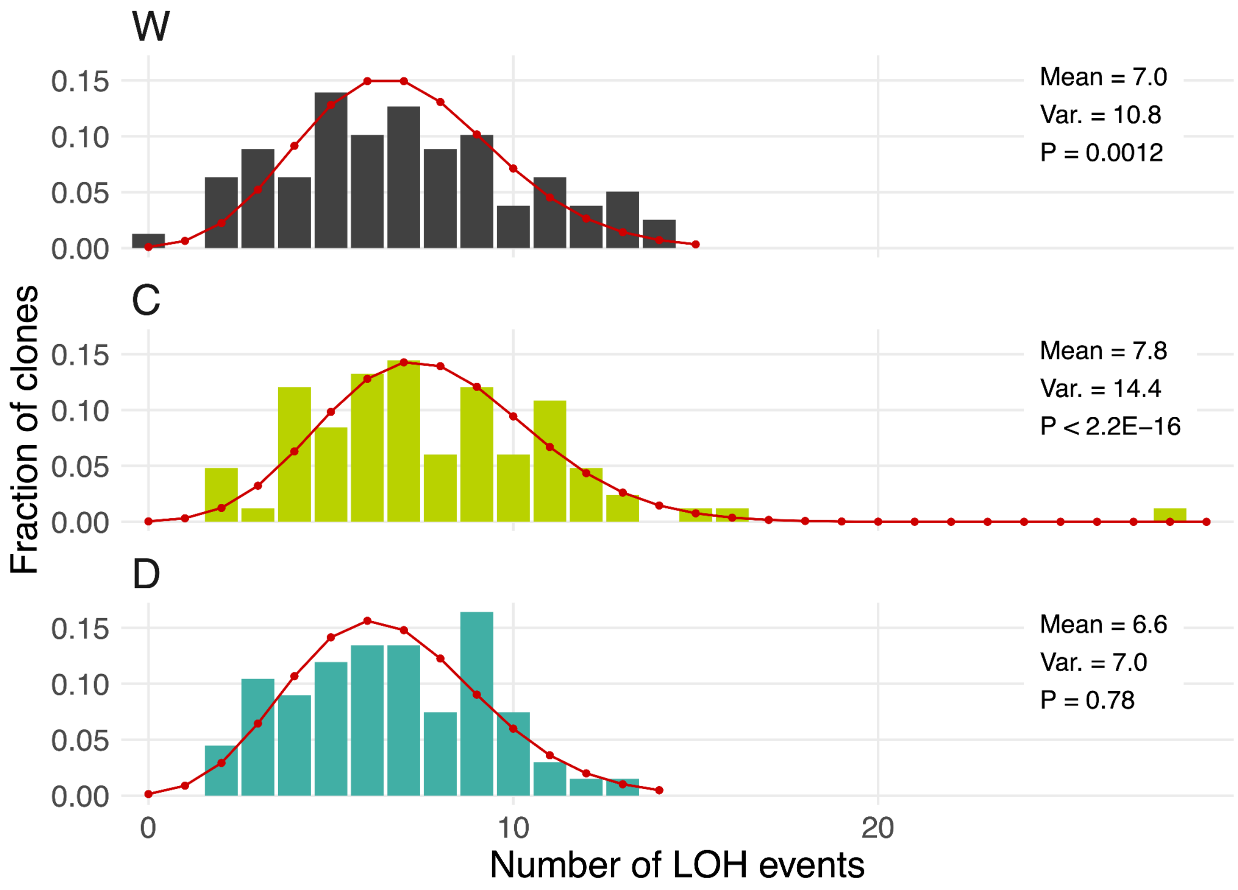

Figure S4. The distribution of LOH events across clones. The best-fit Poisson distribution is shown in red for each strain. *P*-value is calculated for the best-fit Poisson distribution based on the χ^2 goodness-of-fit test.

bioRxiv preprint doi: https://doi.org/10.1101/2023.11.28.569142; this version posted July 7, 2025. The copyright holder for this preprint (which was not certified by peer review) is the author/funder, who has granted bioRxiv a license to display the preprint in perpetuity. It is made available under aCC-BY 4.0 International license.

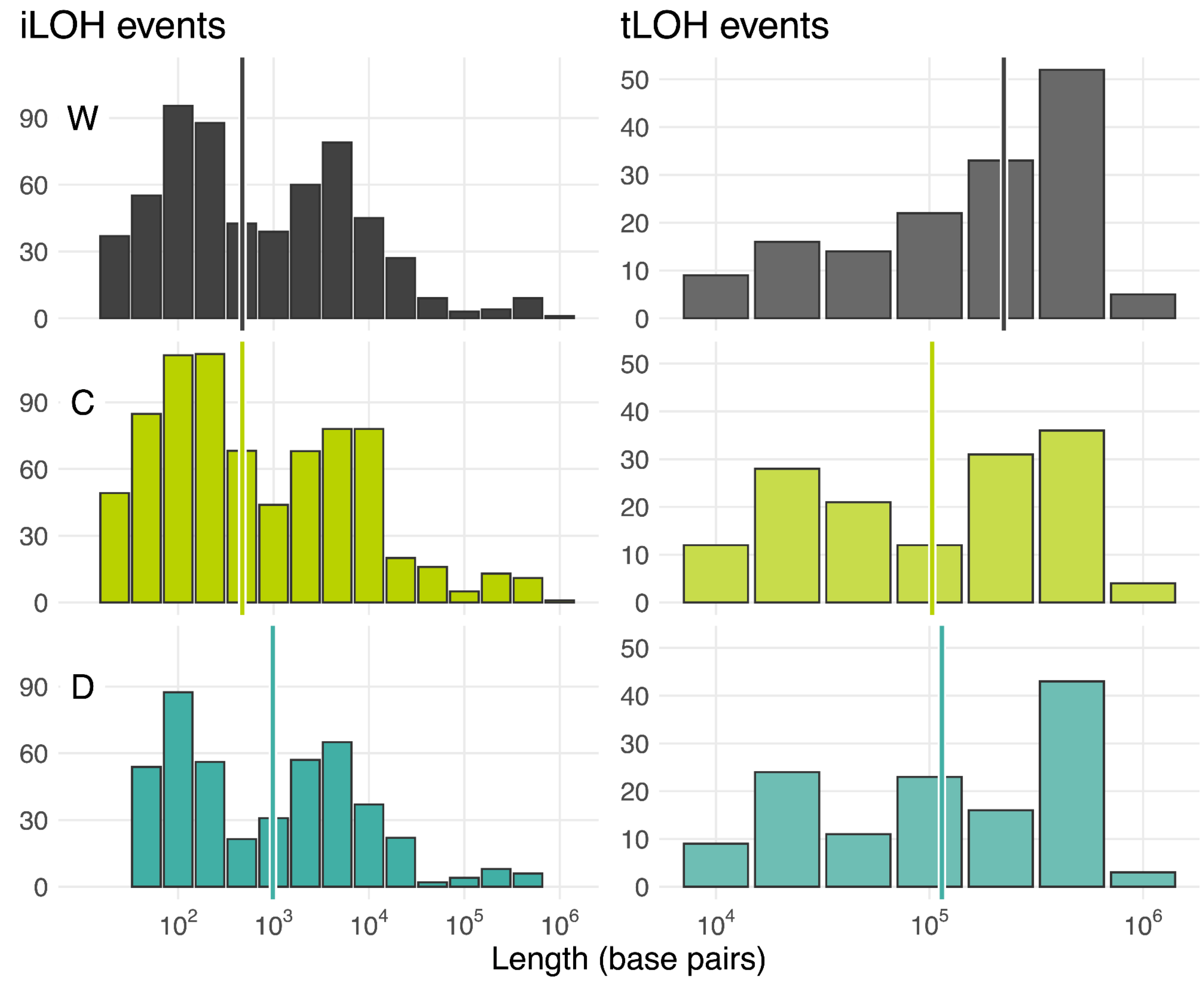

Figure S5. iLOH and tLOH length distributions across the three strains.

bioRxiv preprint doi: https://doi.org/10.1101/2023.11.28.569142; this version posted July 7, 2025. The copyright holder for this preprint (which was not certified by peer review) is the author/funder, who has granted bioRxiv a license to display the preprint in perpetuity. It is made available under aCC-BY 4.0 International license.

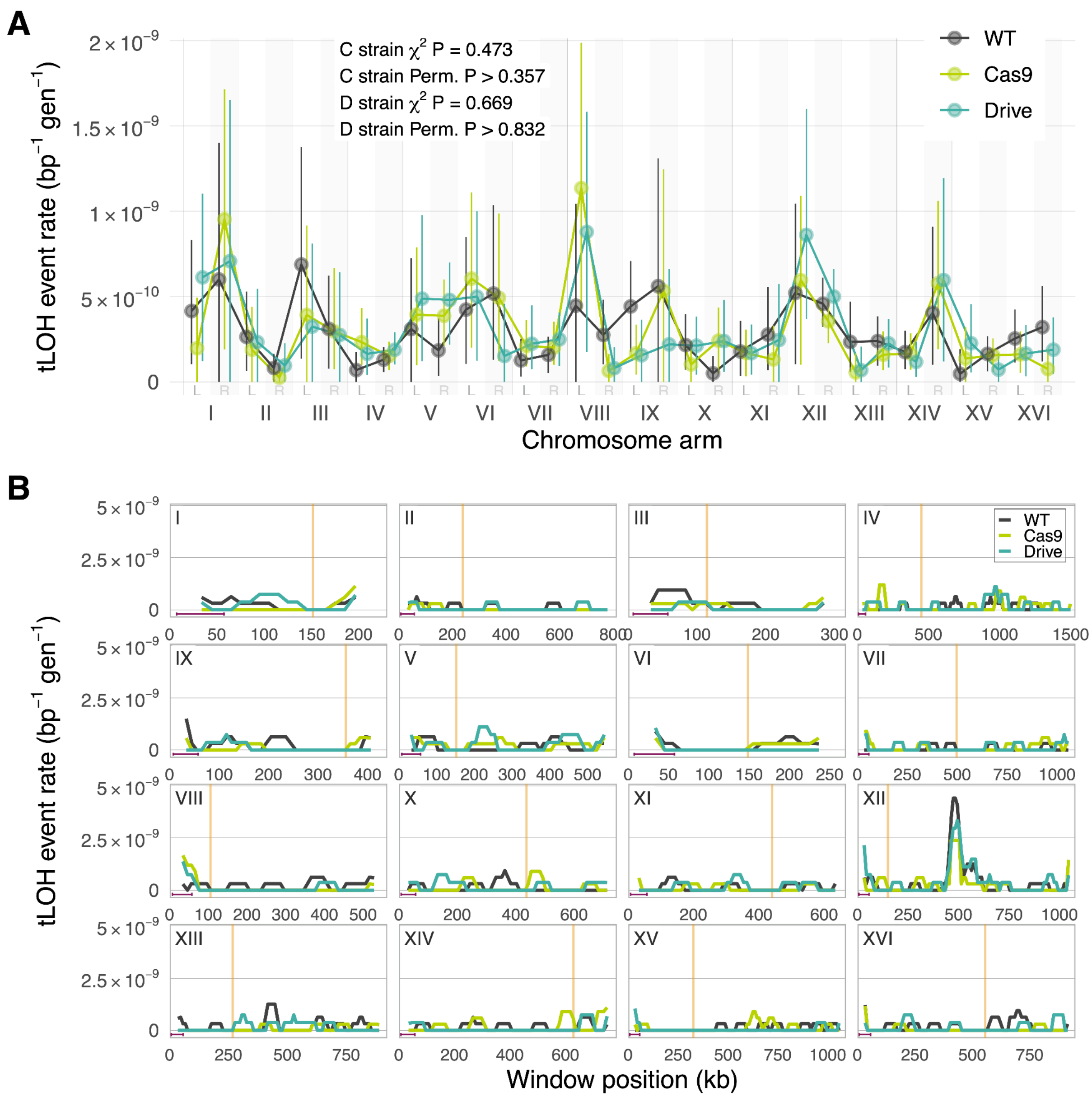

Figure S6. Distribution of tLOH event rates across the genome. Same as Figure 3, but for tLOH events.

bioRxiv preprint doi: https://doi.org/10.1101/2023.11.28.569142; this version posted July 7, 2025. The copyright holder for this preprint (which was not certified by peer review) is the author/funder, who has granted bioRxiv a license to display the preprint in perpetuity. It is made available under aCC-BY 4.0 International license.

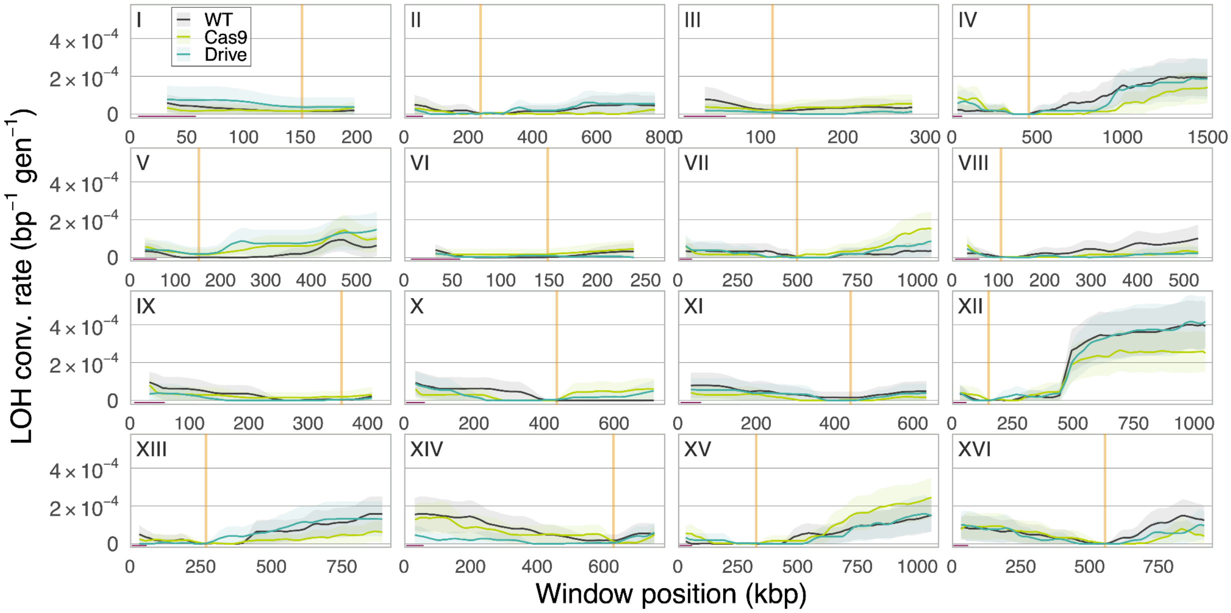

Figure S7. Distribution of LOH conversion rates across the genome. Each line shows the LOH conversion rate estimated within 50 kb sliding windows at 10 kb step sizes. Ribbons indicate 95% bootstrap confidence intervals. Maroon line segments are 50 kb scale bars, orange vertical lines are centromeres.

bioRxiv preprint doi: https://doi.org/10.1101/2023.11.28.569142; this version posted July 7, 2025. The copyright holder for this preprint (which was not certified by peer review) is the author/funder, who has granted bioRxiv a license to display the preprint in perpetuity. It is made available under aCC-BY 4.0 International license.

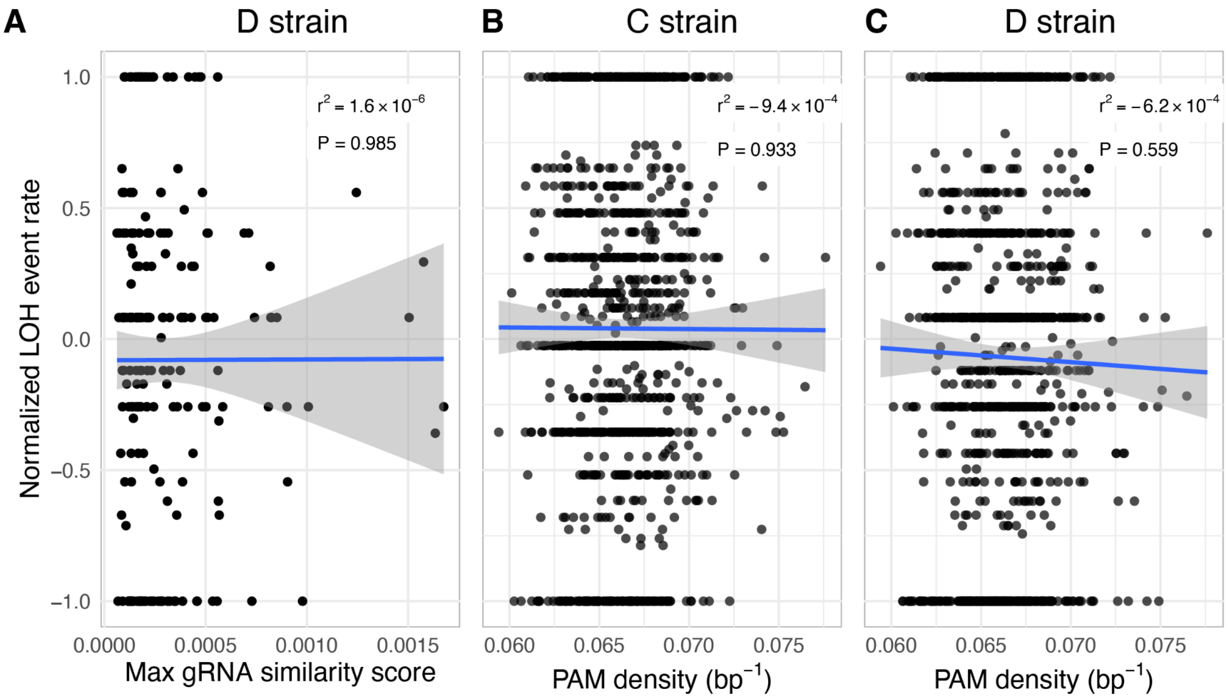

Figure S8. Correlation between the LOH event rate and sequence features across 50 kb genomic windows. Each point represents a 50 kb window in the genome. LOH event rates for C and D strains are normalized to the W rate in the same window as described in Section "Comparison of local LOH rates across strains" in the Materials and Methods. **A.** Normalized rate is plotted against the maximum similarity score between the PAM-adjacent genomic sequences and the gRNA found in a window. **B, C.** Normalized rate is plotted against the density of PAM sites in a window. For all panels, each point is a genomic window, blue lines are linear regressions, gray areas represent 95% confidence intervals.
