# Supplementary material for: Upper Bound on the Mutational Burden Imposed by a CRISPR-Cas9 Gene-Drive Element
Source: bioRxiv. 2023 Nov 29:2023.11.28.569142. Preprint. [Version 1] doi: 10.1101/2023.11.28.569142 (PMC10705488; doi:10.1101/2023.11.28.569142)
Supplement: Supplementary file 1 [file NIHPP2023.11.28.569142V1-supplement-1.pdf]

bioRxiv preprint doi: https://doi.org/10.1101/2023.11.28.569142; this version posted November 29, 2023. The copyright holder for this preprint (which was not certified by peer review) is the author/funder, who has granted bioRxiv a license to display the preprint in perpetuity. It is made available under aCC-BY 4.0 International license.

Supplemental Figures and Tables

Supplementary Table 1. Expected and observed LOH counts and rates for the 16 genomic chromosomes.

		Total			W Strain			C Strain			D Strain		
Chr	Chr length (bp)	Expected LOH	Total LOH	LOH event rate ($\times10^{-10}$)[†]	Expected LOH	Total LOH	LOH event rate ($\times10^{-10}$)[†]	Expected LOH	Total LOH	LOH event rate ($\times10^{-10}$)[†]	Expected LOH	Total LOH	LOH event rate ($\times10^{-10}$)[†]
I	230218	23.9	35	8.48 ± 3.15	7.2	9	3.24 ± 2.16	8.7	17	5.47 ± 2.90	6.1	9	3.80 ± 2.53
II	813184	84.3	82	5.63 ± 1.10	25.3	20	2.04 ± 1.02	30.7	32	2.91 ± 1.00	21.6	26	3.11 ± 1.07
III	316620	32.8	66	11.63 ± 2.64	9.8	13	3.41 ± 1.83	12.0	14	3.27 ± 1.64	8.4	9	2.76 ± 1.84
IV	1531933	158.9	93	3.39 ± 0.66	47.6	30	1.62 ± 0.65	57.9	39	1.89 ± 0.58	40.7	22	1.40 ± 0.63
V	576874	59.8	67	6.48 ± 1.45	17.9	22	3.16 ± 1.29	21.8	21	2.70 ± 1.28	15.3	22	3.71 ± 1.35
VI	270161	28.0	24	4.96 ± 2.06	8.4	10	3.07 ± 1.84	10.2	6	1.64 ± 1.37	7.2	8	2.88 ± 2.16
VII	1090940	113.1	105	5.37 ± 1.07	33.9	25	1.90 ± 0.83	41.2	44	2.99 ± 1.02	29.0	22	1.96 ± 0.80
VIII	562643	58.4	71	7.04 ± 1.69	17.5	26	3.83 ± 1.62	21.3	28	3.69 ± 1.32	15.0	17	2.94 ± 1.21
IX	439888	45.6	116	14.71 ± 2.54	13.7	34	6.41 ± 1.89	16.6	38	6.40 ± 1.68	11.7	29	6.41 ± 2.21
X	745751	77.3	40	2.99 ± 1.05	23.2	6	0.67 ± 0.56	28.2	17	1.69 ± 0.89	19.8	17	2.22 ± 1.30
XI	666816	69.2	41	3.43 ± 1.00	20.7	14	1.74 ± 0.99	25.2	18	2.00 ± 0.78	17.7	9	1.31 ± 0.87
XII	1078177	111.8	133	6.88 ± 1.19	33.5	41	3.15 ± 0.92	40.7	48	3.30 ± 0.82	28.7	38	3.43 ± 1.08
XIII	924431	95.9	76	4.59 ± 1.21	28.7	28	2.51 ± 0.81	34.9	29	2.32 ± 0.88	24.6	14	1.47 ± 0.74
XIV	784333	81.3	103	7.33 ± 1.56	24.4	26	2.75 ± 1.16	29.6	45	4.25 ± 1.23	20.9	24	2.97 ± 1.24
XV	1091291	113.2	115	5.88 ± 1.02	33.9	37	2.81 ± 0.91	41.2	34	2.31 ± 0.81	29.0	33	2.94 ± 0.89
XVI	948066	98.3	85	5.00 ± 1.12	29.5	34	2.97 ± 1.05	35.8	26	2.03 ± 1.02	25.2	22	2.26 ± 0.92
Genome	12071326	NA	1152	2.66 ± 0.17	387.4	375	2.58 ± 0.34	433.9	456	2.80 ± 0.31	330.6	321	2.59 ± 0.37

[†] per base-pair per generation ± 95% bootstrap confidence interval, accounting for diploid genome

bioRxiv preprint doi: https://doi.org/10.1101/2023.11.28.569142; this version posted November 29, 2023. The copyright holder for this preprint (which was not certified by peer review) is the author/funder, who has granted bioRxiv a license to display the preprint in perpetuity. It is made available under aCC-BY 4.0 International license.

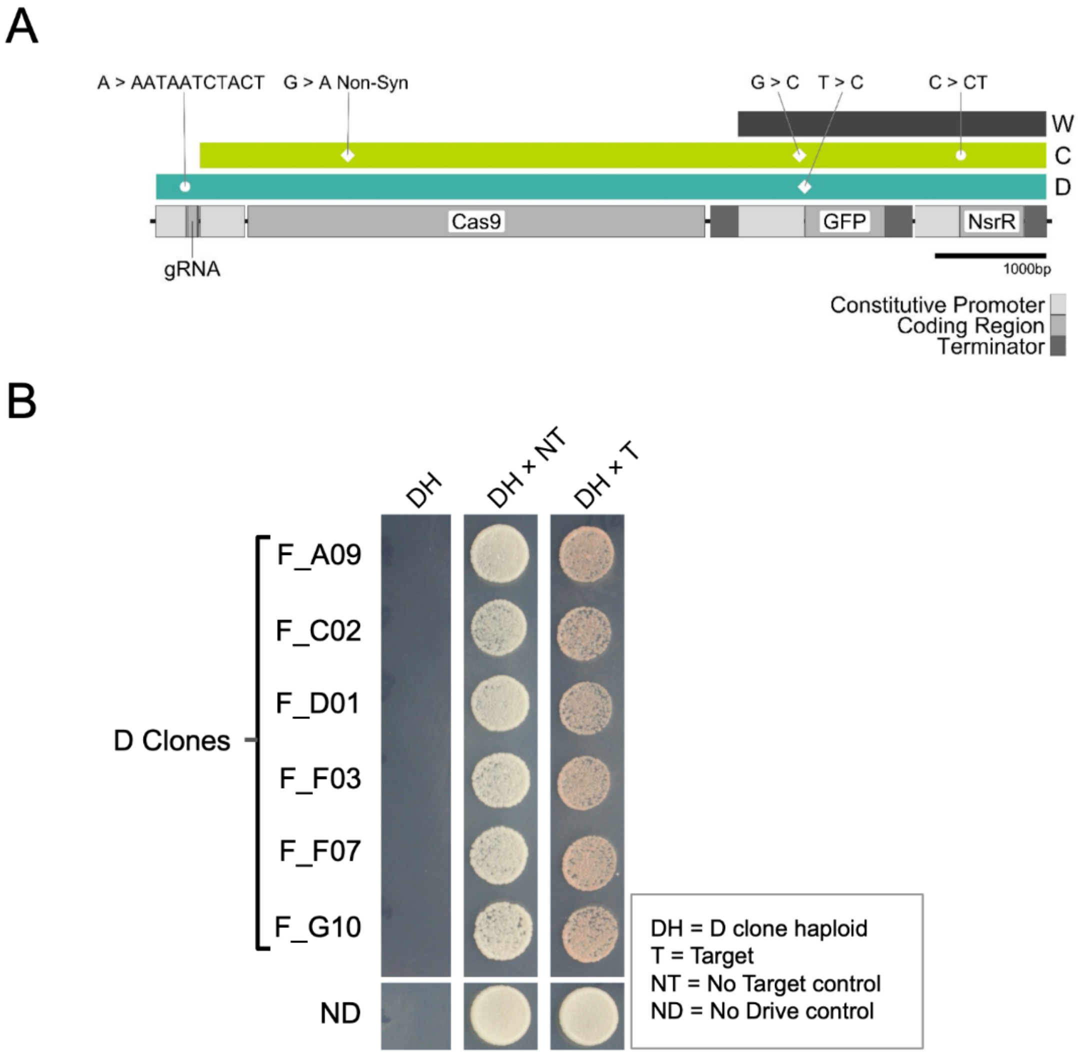

Supplementary Figure 1. CCGD remains active throughout the MA experiment. A. Mutations accumulated in the drive constructs for each strain. The construct map shows the position of each component and the three colored bars above represent the portion of the construct integrated into each strain. Diamonds indicate point mutations and circles indicate indels. **B.** All D end-point clones tested retain drive activity. A haploid offspring of each tested D clone (rows) was mated with a tester strain with the sequence targeted by the gRNA (T) and with a tester strain without the target sequence (NT). Mated and unmated liquid cultures were spot plated on CSM + 5FOA agar, then replica plated on CSM + G418 agar for diploid selection and incubated for 48 hours. A successful disruption of the ADE2 gene by the CCGD results in red colony coloration (DiCarlo et al. 2015).

bioRxiv preprint doi: https://doi.org/10.1101/2023.11.28.569142; this version posted November 29, 2023. The copyright holder for this preprint (which was not certified by peer review) is the author/funder, who has granted bioRxiv a license to display the preprint in perpetuity. It is made available under aCC-BY 4.0 International license.

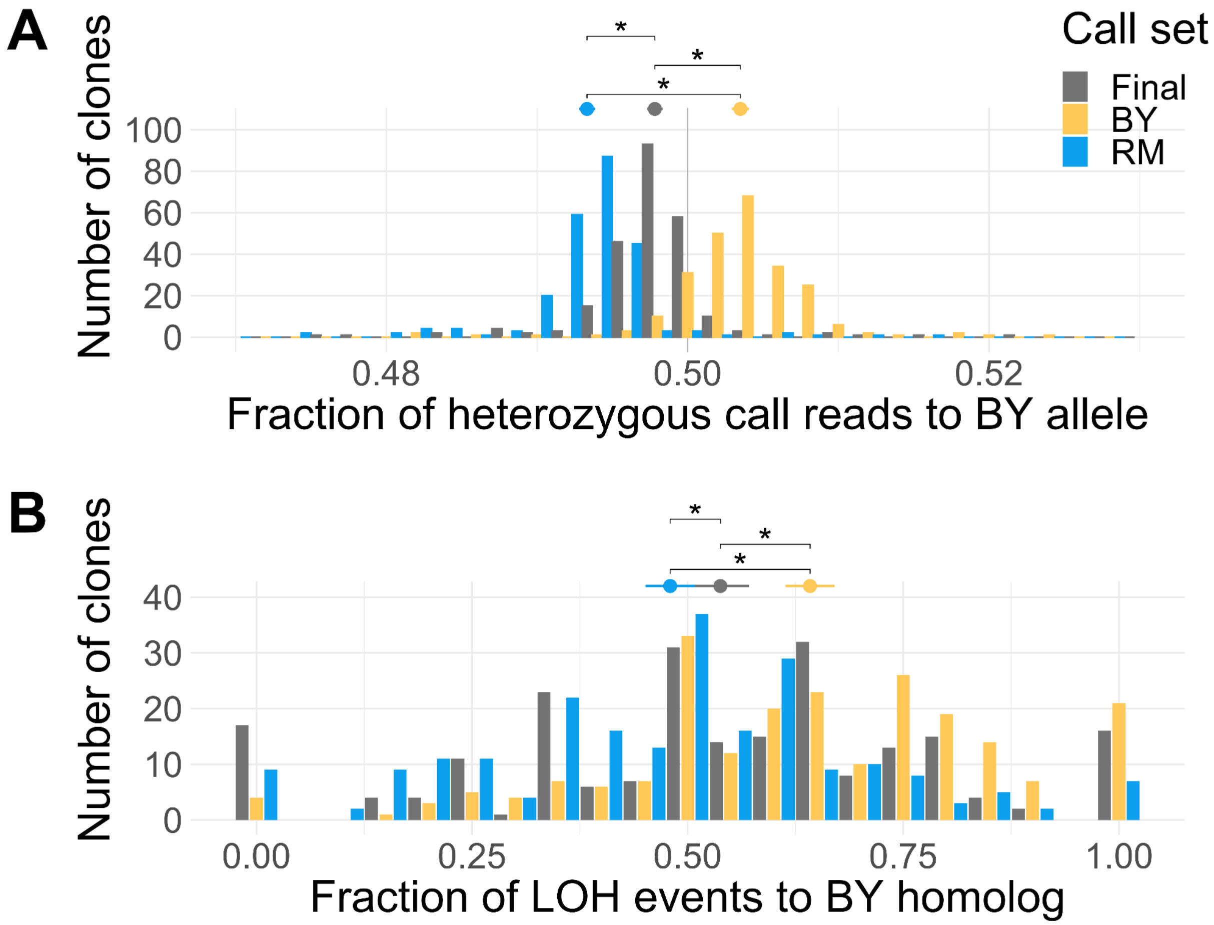

Supplementary Figure 2. Choice of the reference sequence introduces biases into read mapping and LOH detection. A. The mean fraction of reads supporting the BY allele across all heterozygous calls in each clone when called against each reference sequence (BY and RM) and after undergoing reference symmetric calling (Final) (see Methods). Filled circles indicate the means and lines the 95% confidence intervals of the means for each distribution. All comparisons resulted in $P < 0.001$ with Mann-Whitney test after Benjamini-Hochberg correction. **B.** The fraction of LOH events converted to the BY homolog was calculated for each end-point clone for each of the call sets. Filled circles indicate the means and lines the 95% confidence intervals of the means for each distribution. All comparisons resulted in $P < 0.001$ with Mann-Whitney test after Benjamini-Hochberg correction.

bioRxiv preprint doi: https://doi.org/10.1101/2023.11.28.569142; this version posted November 29, 2023. The copyright holder for this preprint (which was not certified by peer review) is the author/funder, who has granted bioRxiv a license to display the preprint in perpetuity. It is made available under aCC-BY 4.0 International license.

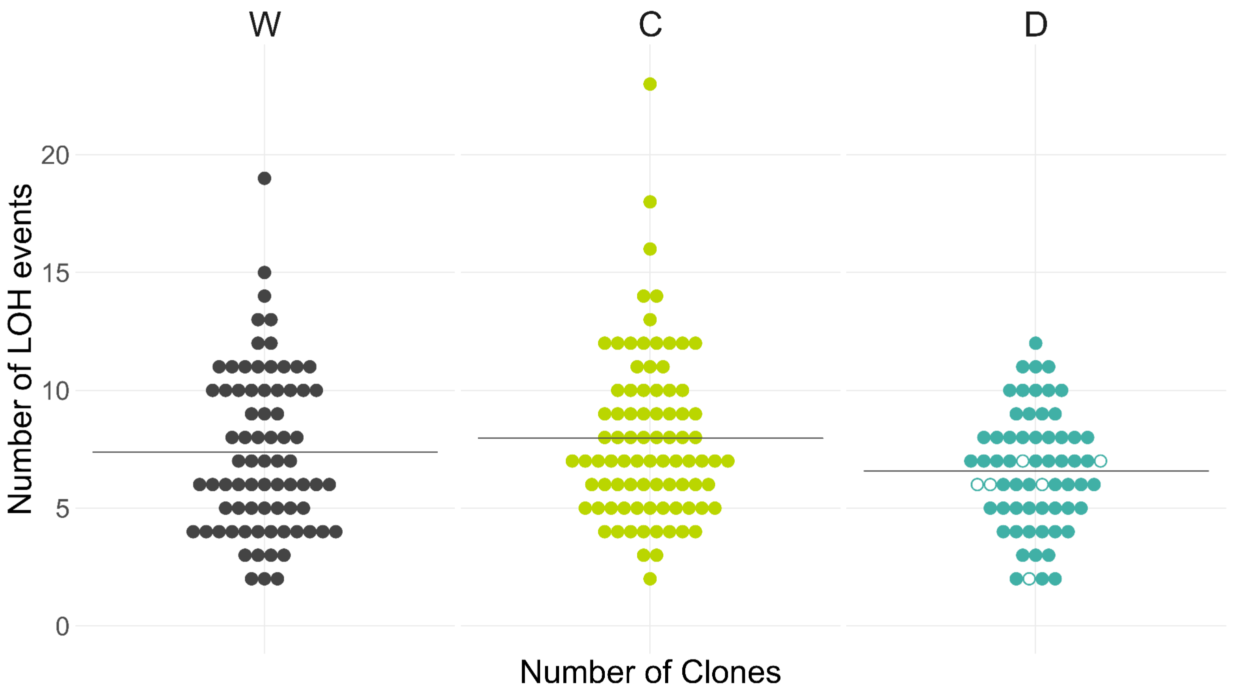

Supplementary Figure 3. Distributions of counts of putative LOH events among end-point clones prior to false homozygous call error correction. Hollow circles indicate clones tested for drive activity. Horizontal lines indicate the mean number of LOH events per clone.

bioRxiv preprint doi: https://doi.org/10.1101/2023.11.28.569142; this version posted November 29, 2023. The copyright holder for this preprint (which was not certified by peer review) is the author/funder, who has granted bioRxiv a license to display the preprint in perpetuity. It is made available under aCC-BY 4.0 International license.

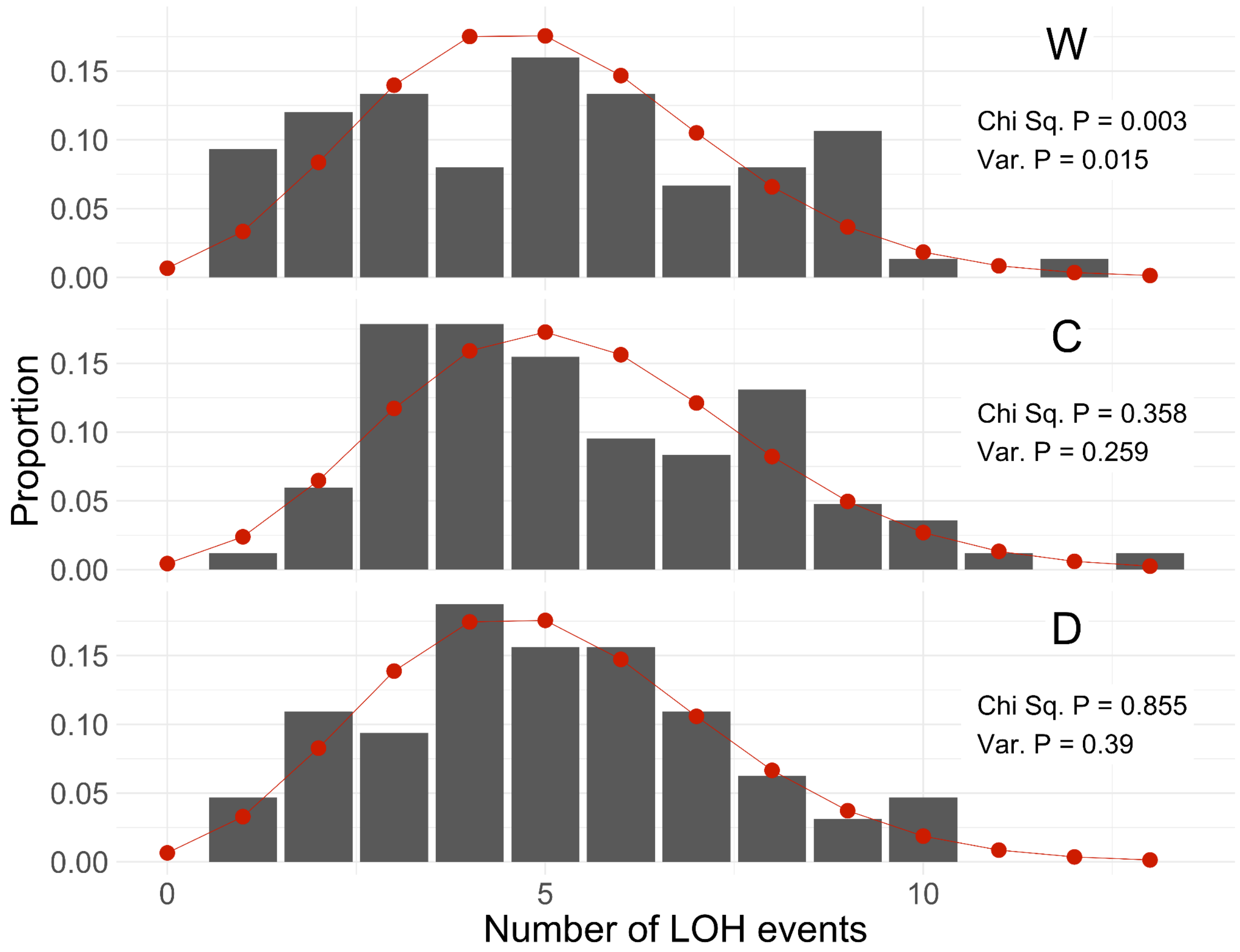

Supplementary Figure 4. Distributions of LOH events per clone. The best-fit Poisson distribution is shown in red. *P*-values are calculated from χ^2 goodness-of-fit and permutation tests of the differences in variances between the observed distributions and their corresponding Poisson distributions.

bioRxiv preprint doi: https://doi.org/10.1101/2023.11.28.569142; this version posted November 29, 2023. The copyright holder for this preprint (which was not certified by peer review) is the author/funder, who has granted bioRxiv a license to display the preprint in perpetuity. It is made available under aCC-BY 4.0 International license.

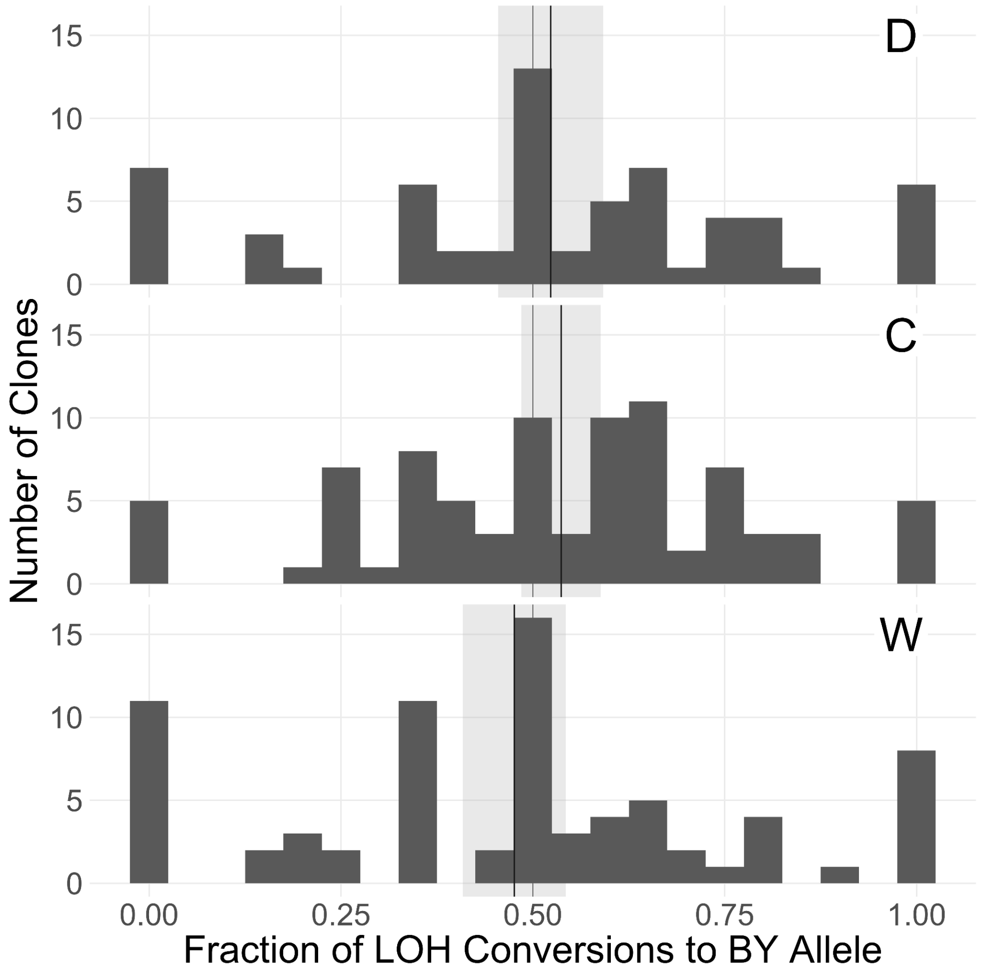

Supplementary Figure 5. LOH conversions do not exhibit homolog bias in any of the three strains. The fraction of LOH events that were converted to the BY homolog was calculated for each clone. Vertical lines indicate the mean BY bias and the shading indicates the 95% confidence intervals of the mean.

bioRxiv preprint doi: https://doi.org/10.1101/2023.11.28.569142; this version posted November 29, 2023. The copyright holder for this preprint (which was not certified by peer review) is the author/funder, who has granted bioRxiv a license to display the preprint in perpetuity. It is made available under aCC-BY 4.0 International license.

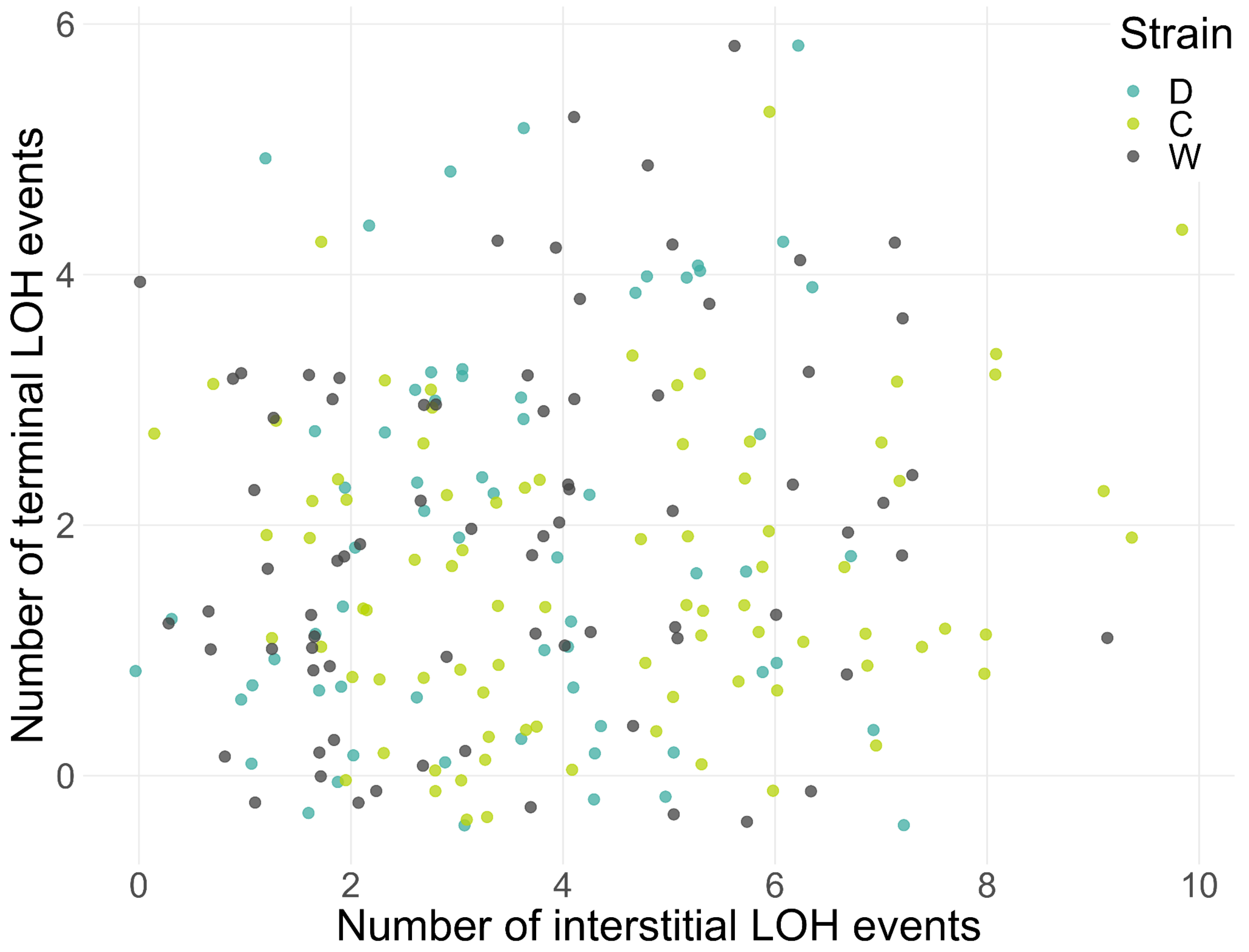

Supplementary Figure 6. Comparison between interstitial LOH and terminal LOH event accumulation shows a lack of correlation. Each dot represents an end-point clone and color indicates the strain.

bioRxiv preprint doi: https://doi.org/10.1101/2023.11.28.569142; this version posted November 29, 2023. The copyright holder for this preprint (which was not certified by peer review) is the author/funder, who has granted bioRxiv a license to display the preprint in perpetuity. It is made available under aCC-BY 4.0 International license.

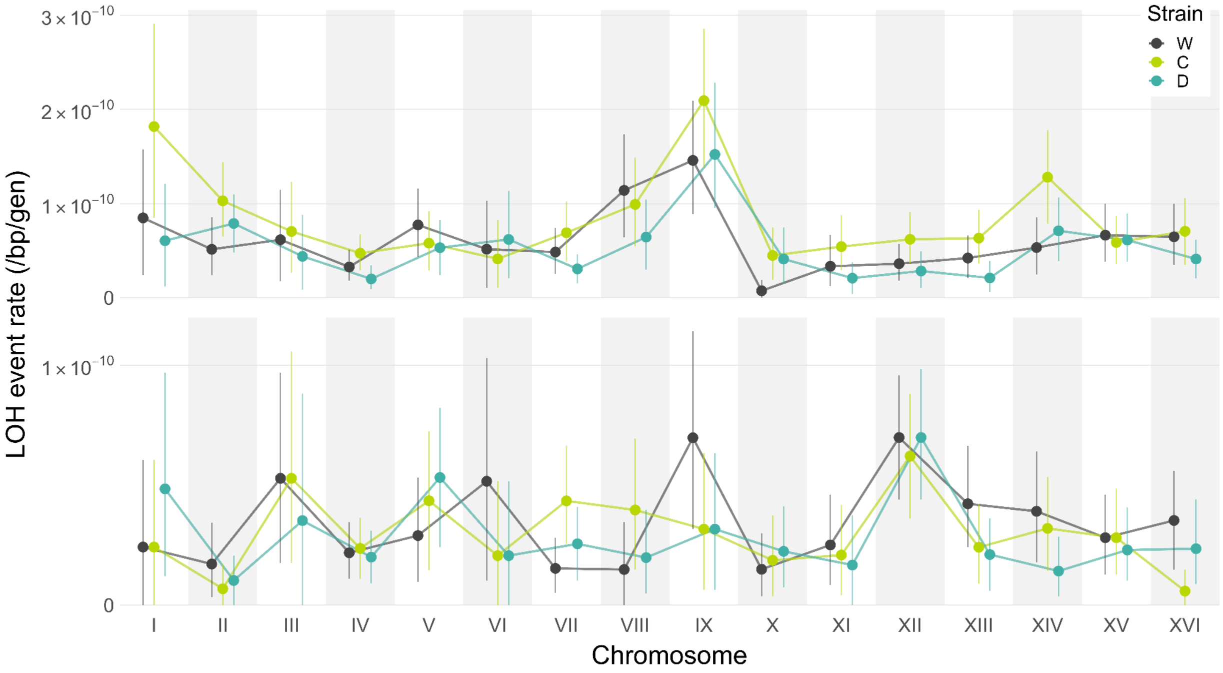

Supplementary Figure 7. Distributions of iLOH and tLOH event counts across chromosomes. Notations are as in Figure 4.

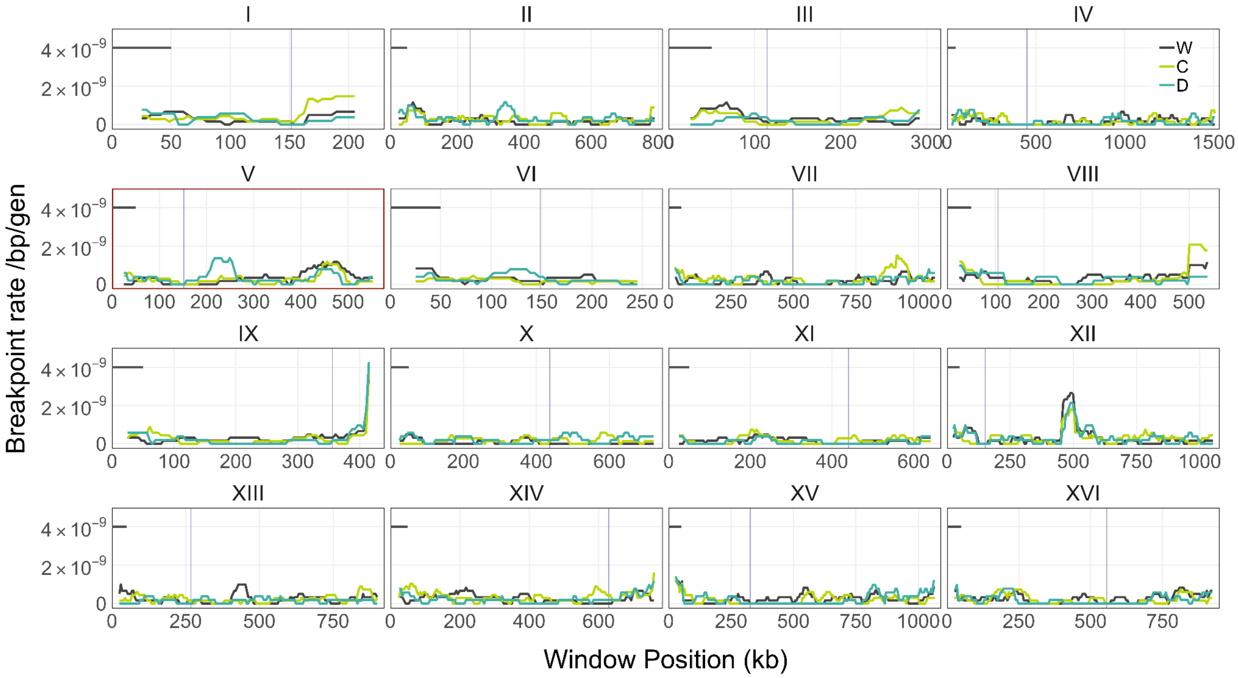

Supplementary Figure 8. 50kb sliding window estimates of breakpoint rates with full y-axis scale. Each line shows the breakpoint rate estimated per 50 kb sliding window with a 5 kb step size, as in **Figure 5**. (see Methods for details).

bioRxiv preprint doi: https://doi.org/10.1101/2023.11.28.569142; this version posted November 29, 2023. The copyright holder for this preprint (which was not certified by peer review) is the author/funder, who has granted bioRxiv a license to display the preprint in perpetuity. It is made available under aCC-BY 4.0 International license.

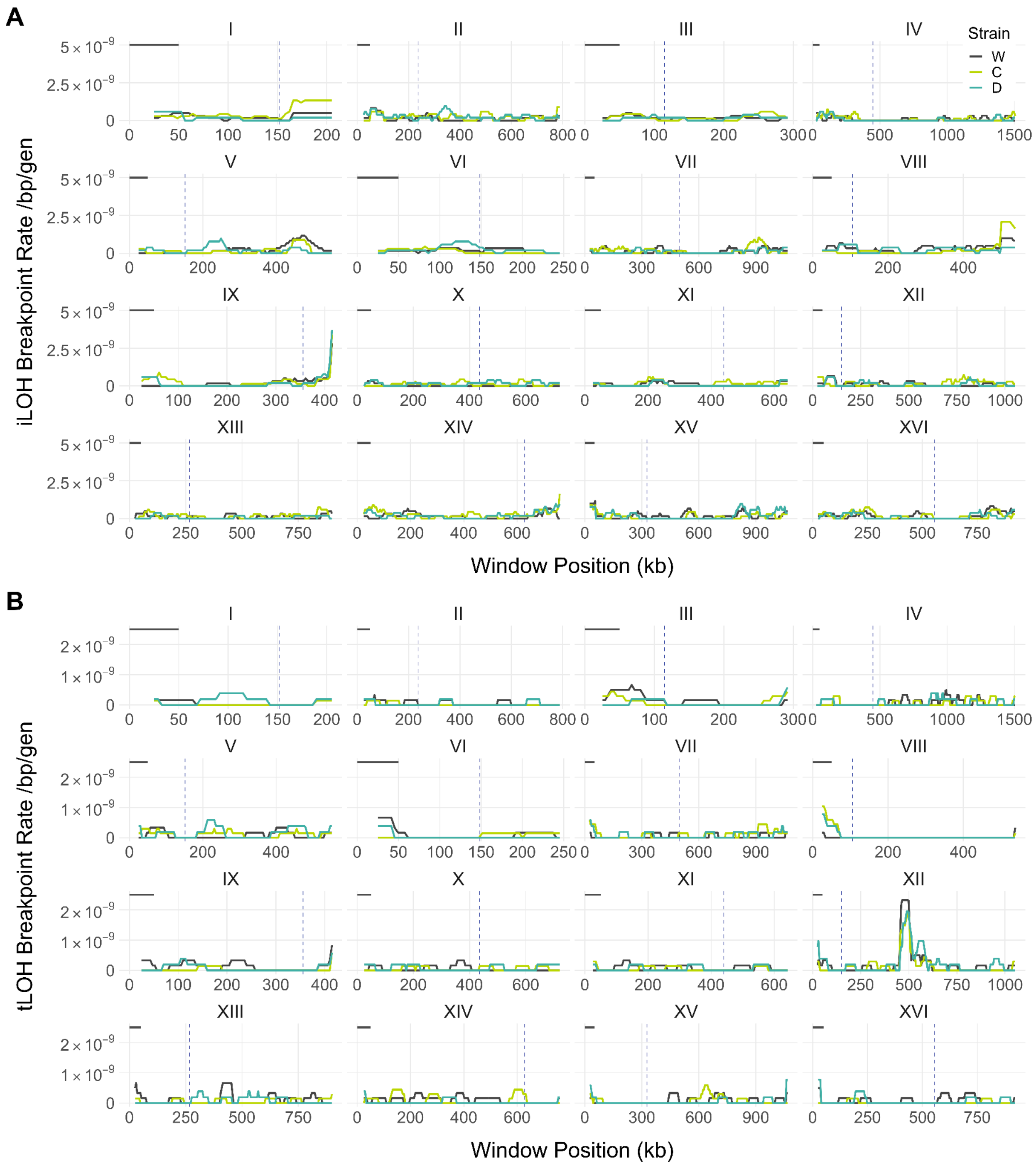

Supplementary Figure 9. 50kb sliding window estimates of breakpoint rates for iLOH and tLOH events. A. Sliding window interstitial LOH breakpoint rates as in **Figure 5. B.** Sliding window terminal LOH breakpoint rates as in **Figure 5.**

bioRxiv preprint doi: https://doi.org/10.1101/2023.11.28.569142; this version posted November 29, 2023. The copyright holder for this preprint (which was not certified by peer review) is the author/funder, who has granted bioRxiv a license to display the preprint in perpetuity. It is made available under aCC-BY 4.0 International license.

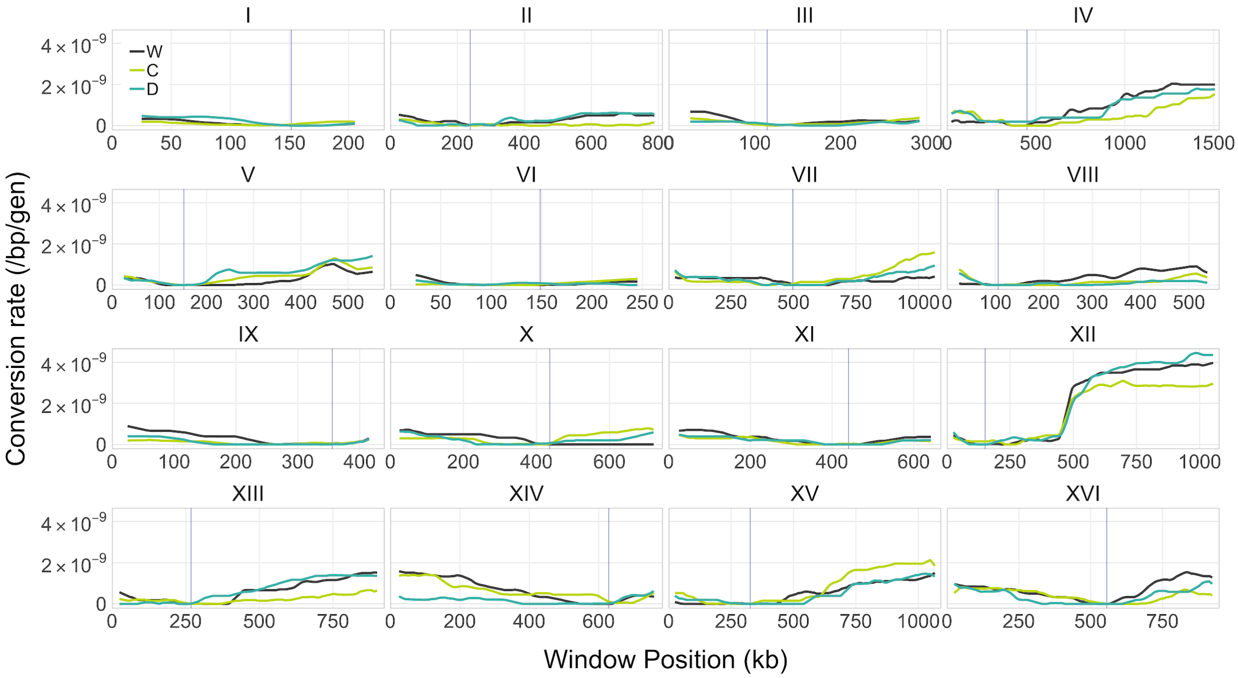

Supplementary Figure 10. 50kb sliding window estimates of LOH rates. Each line shows the LOH rate estimated per 50 kb sliding window with a 5 kb step size (see Methods for details). Line segments are 50kb scale bars.

bioRxiv preprint doi: https://doi.org/10.1101/2023.11.28.569142; this version posted November 29, 2023. The copyright holder for this preprint (which was not certified by peer review) is the author/funder, who has granted bioRxiv a license to display the preprint in perpetuity. It is made available under aCC-BY 4.0 International license.

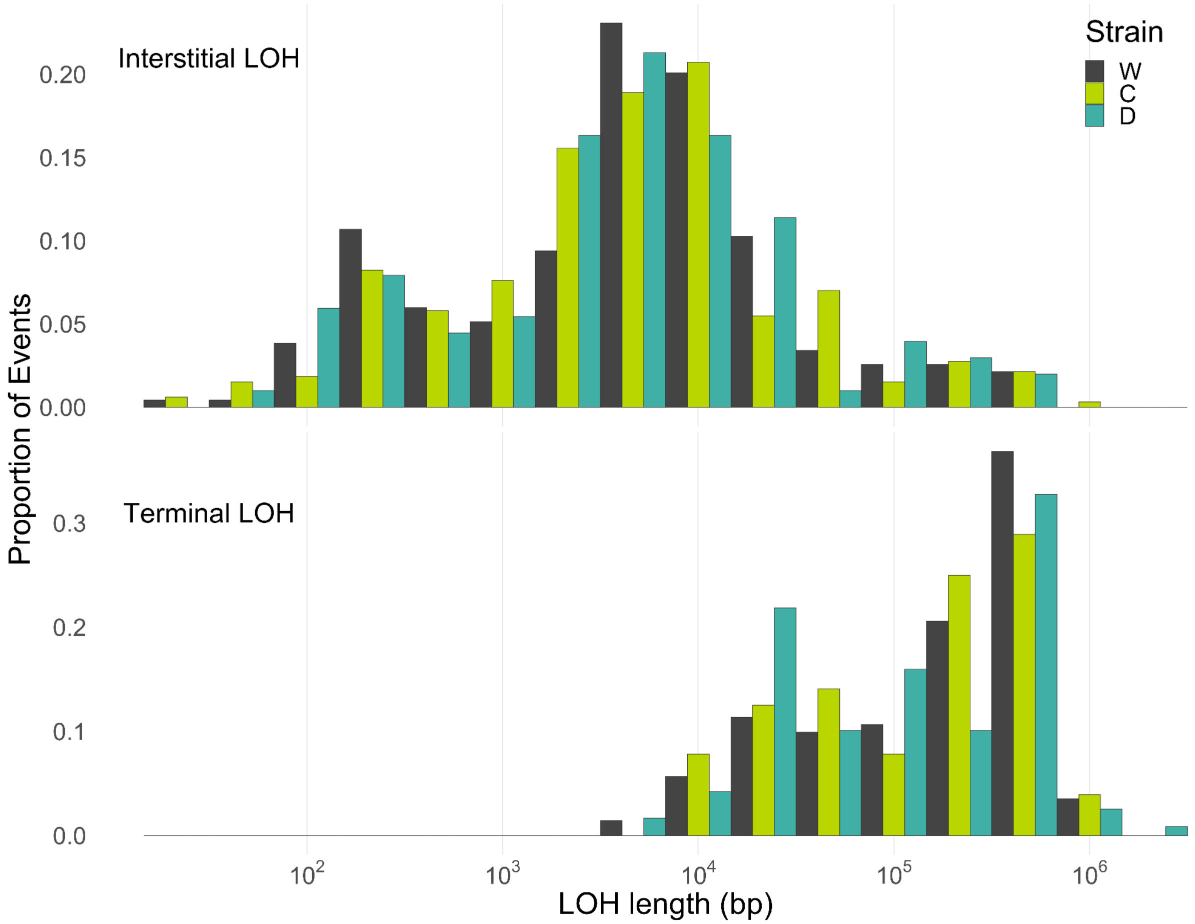

Supplementary Figure 11. iLOH and tLOH length distributions for the three strains. For each LOH type, the proportion of events was tallied for each log-scaled length bins.
